# Keys to world Charipinae (Hymenoptera, Cynipoidea, Figitidae)

**DOI:** 10.3897/zookeys.822.30151

**Published:** 2019-02-06

**Authors:** Mar Ferrer-Suay, Jesús elfa, Juli Pujade-Villar

**Affiliations:** 1 Universitat de València, Facultat de Ciències Biològiques, Departament de Zoologia, Campus de Burjassot-Paterna, Dr. Moliner 50, 46100 Burjassot (València), Spain; 2 Universitat de Barcelona, Facultat de Biologia, Departament de Biologia Animal. Avda, Diagonal 645, 08028-Barcelona, Spain

**Keywords:** *
Alloxysta
*, *
Apocharips
*, Charipinae, *
Dilapothor
*, *
Dilyta
*, key, *
Lobopterocharips
*, *
Lytoxysta
*, *
Phaenoglyphis
*, *
Thoreauana
*

## Abstract

Eight genera of Charipinae are defined, keyed out, and illustrated. Keys for all charipine species within each valid genus, including *Alloxysta*, *Apocharips*, *Dilyta*, *Phaenoglyphis*, and *Thoreauana*, are presented, except for *Dilapothor*, *Lobopterocharips*, and *Lytoxysta*, which are monotypic. Figures are provided to show the diagnostic morphological features as used in the keys.

## Introduction

Hymenopteran parasitoids are one of the most important groups of insects in pest control. However, their use in controlling pests is usually difficult because they are impossible to identify at species level; therefore, their employment in pest control is limited. For this reason, the focus of our study is the basic taxonomy of the many hymenopteran groups that are still unknown. This has been our main aim over the last few years.

The Figitidae (Hymenoptera, Cynipoidea) are biologically characterised as parasitoids of the larvae of other insects, principally Diptera and Cyclorrapha (Ronquist 1999), except for the subfamily Charipinae, which is parasitoids of Hemiptera. The members of this subfamily are hyperparasitoids of aphids via Aphidiinae (Hymenoptera, Ichneumonoidea, Braconidae) and Aphelininae (Hymenoptera, Chalcidoidea, Aphelinidae), as well as hyperparasitoids of psyllids via Encyrtidae (Hymenoptera, Chalcidoidea) (Fergusson 1986; Menke and Evenhuis 1991).

Eight genera of Charipinae are recognised in the following regions: *Alloxysta* (Cosmopolitan; Förster 1869), *Apocharips* (Palaearctic and Neotropical; Fergusson 1986), *Dilapothor* (Australia; Paretas-Martínez and Pujade-Villar 2006), *Dilyta* (cosmopolitan, except South America and Australia; Förster 1869), *Lobopterocharips* (Nepal; Paretas-Martínez and Pujade-Villar 2007), *Lytoxysta* (North America; Kieffer 1909), *Phaenoglyphis* (cosmopolitan; Förster 1869), and *Thoreauana* (Australia; Girault 1930). The morphological features of the charipine specimens are dramatically reduced, mainly because they are very small (0.8–2.0 mm), and they generally have shiny, smooth bodies. The combination of these features with the large number of described species over the past 150 years has been the reason for the chaotic taxonomy of the Charipinae. This makes the identification of individual species very difficult.

Nowadays, thanks to many studies carried out the correct state of each species has been rectified, and many new species have been described. The taxonomy of the subfamily Charipinae is now organised and clear, due to studies published over the last nine years (e.g. Ferrer-Suay et al. 2013d, e, f, g, h; 2014a, b, 2015; 2018). Additionally, a website has been developed to collect accurate, up-to-date information on the Charipinae (http://www.charipinaedatabase.com)

Following these studies, and taking into account the importance of the Charipinae in ecology, we have prepared clear and easily followed keys for each of the genera of Charipinae. Figures show the diagnostic characters, as used in the keys. These keys will be helpful for those looking to identify charipine species. The analysis of the morphological features of each species has been crucial to elaborate on these keys.

## Material and methods

Specimens were studied using a stereomicroscope (NIKON SMZ-1) and an environmental scanning electron microscope (FEI Quanta 200 ESEM) at the scientific technical services of the University of Barcelona. The field-emission gun environmental scanning electron microscope was used for high-resolution imaging without the need to gold-coat the specimens.

Type materials of each species of Charipinae have been reviewed, as have many additional specimens from all over the world and deposited in the following institutions:

**CNCI** (Canadian National Collection of Insects, Ottawa, Canada; G. Gibson)

**USNM** (National Museum of Natural History (Smithsonian Institution), Washington, DC, USA; M. Buffington)

**BMNH** (Natural History Museum, London, England; D. Notton)

**MZLU** (Biologiska Museet, Lund, Sweden; R. Danielsson)

**ZSM** (Zoologische Staatssammlung Museum, Munich, Germany; S. Schmidt)

Morphological terms used follow Paretas-Martínez et al. (2007). Measurements and abbreviations include F1–F12, first and subsequent flagellomeres. The width of the forewing radial cell is measured from the margin of the wing to the beginning of the Rs vein. The transfacial line is measured as the distance between the inner margins of compound eyes, measured across the face through the antennal sockets divided by the height of the eye. The malar space is measured by the distance from the lower part of the gena from the mouthparts to the ventral margin of the compound eye, divided by the height of the eye. Females and males of the species have the same characters except where indicated.

## Results

Below are explained the morphological features important for species or genera identification in charipines, according to Ferrer-Suay et al. (2012).

**Figure 1.**
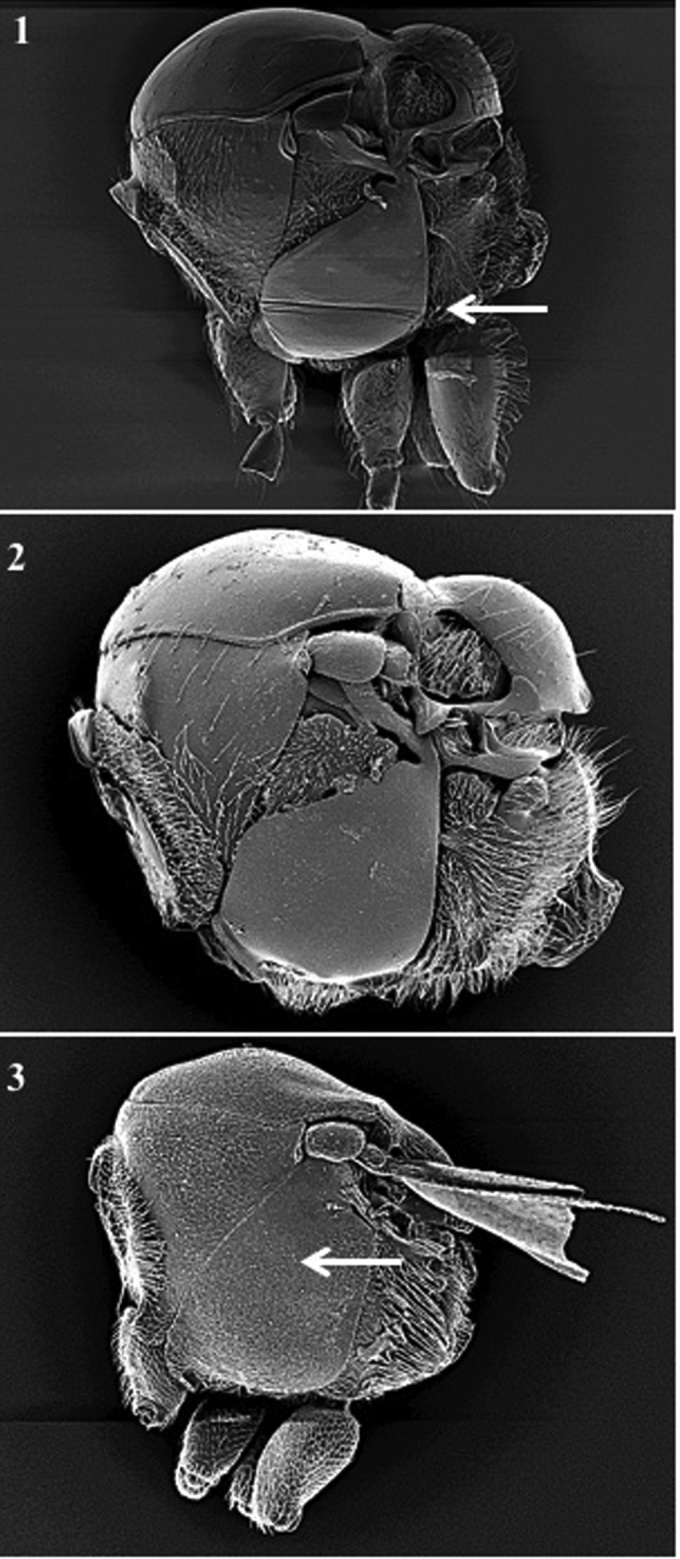
Body surface. *Phaenoglyphis* sp. (**1**); *Alloxysta* sp. (**2**); *Lytoxysta* sp. (**3**).

**Body surface** (Fig. 1)

*Generic characters*. With very fine reticulate sculpture in antennae, head and mesosoma (*Lytoxysta*, Fig. 1[3]) / smooth all other genera (Fig. 1[1, 2]) (except some *Phaenoglyphis* species, which have some very fine imbricate sculpture in scutum).

**Head** (Fig. 2)

**Figure 2.**
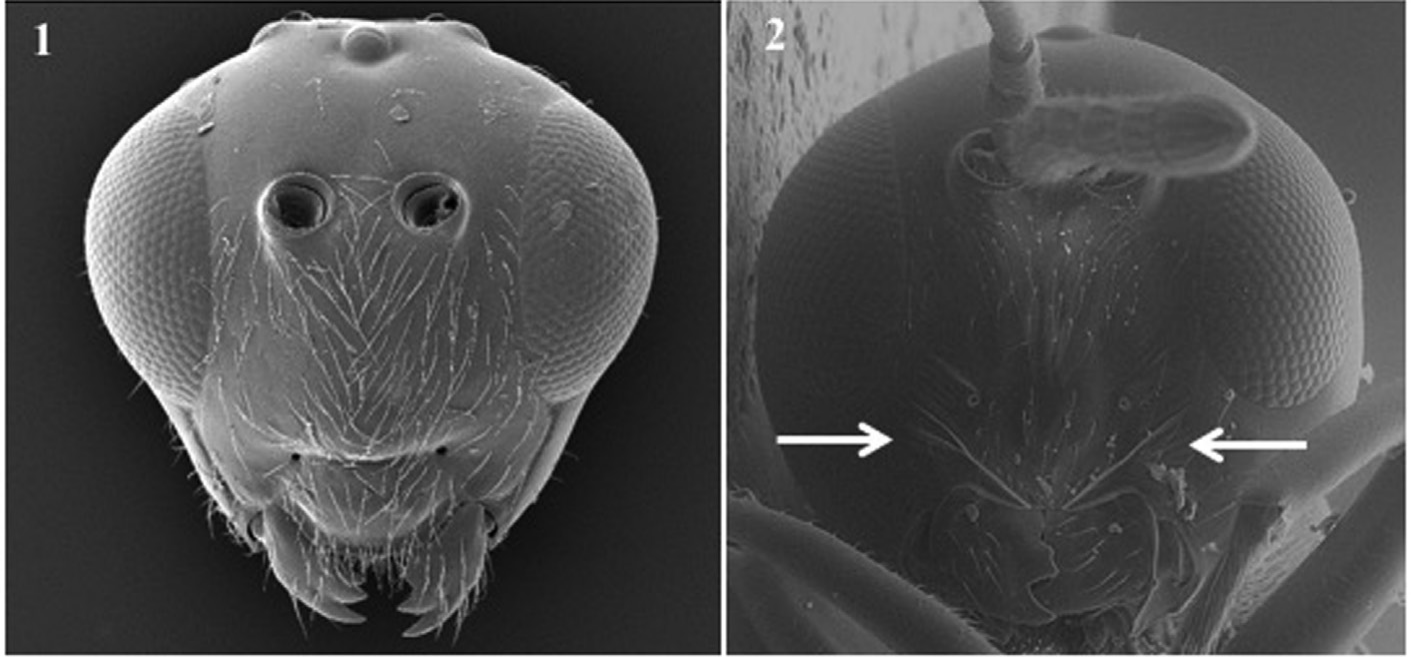
Head. *Phaenoglyphisamericana* (**1**); *Apocharipshansoni* (**2**).

*Specific characters*. Radial carinae on face (only for *Apocharips* species, Fig. 2[2]) / smooth (rest of the genera) (Fig. 2[1]).

**Figure 3.**
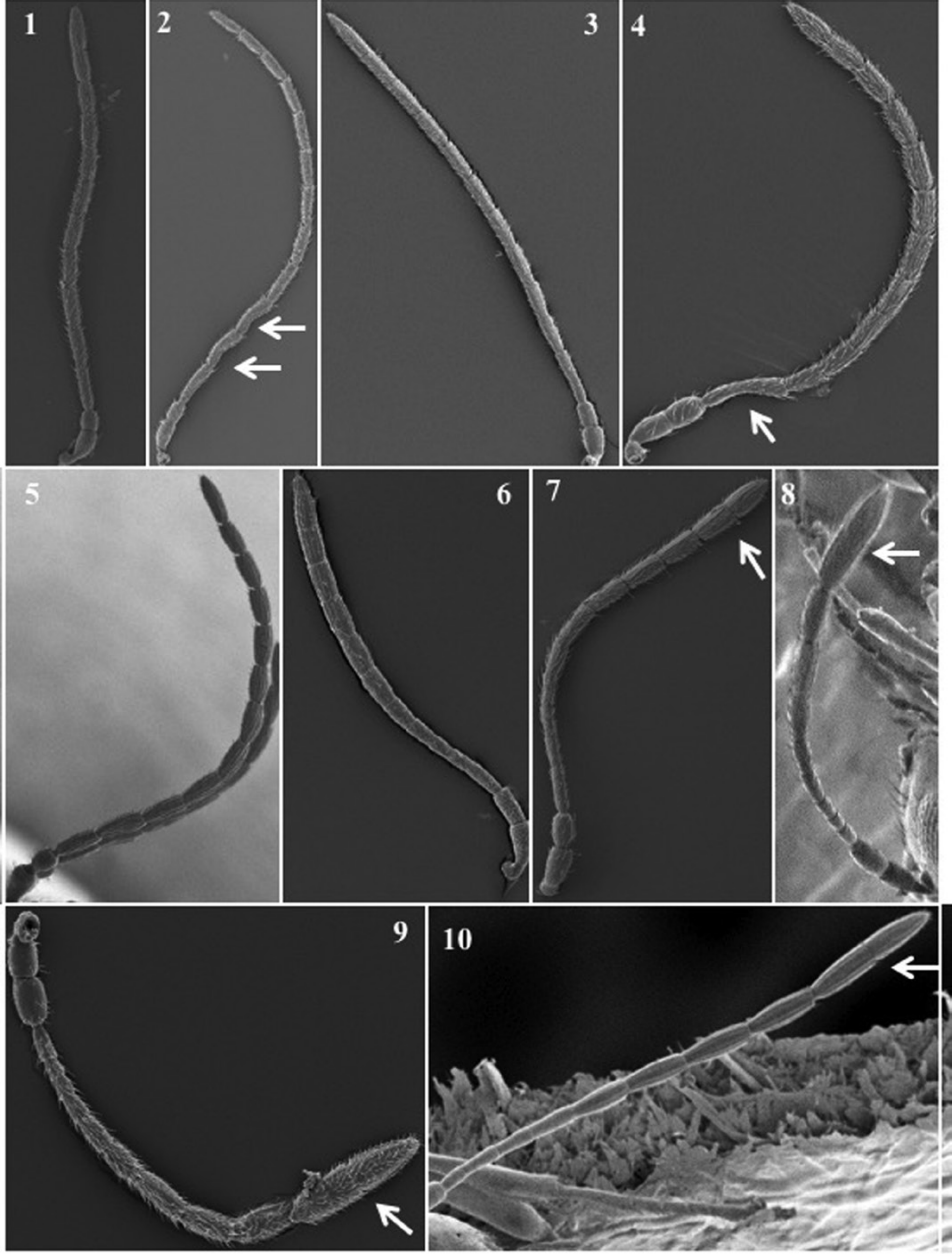
Antenna. *Alloxystavictrix*, female (**1**); *Alloxystavictrix*, male (**2**); *Phaenoglyphisamericana*, female (**3**); *Phaenoglyphisamericana*, male (**4**); *Lobopterocharipsarreplegata*, male (**5**); *Lytoxystabrevipalpis*, female (**6**); *Apocharipstrapezoidea*, female (**7**); *Thoreauanamascagnini*, female (**8**); *Dilytasubclavata*, female (**9**); *Dilapothorcarverae*, female (**10**).

**Antenna** (Fig. 3)

*Generic characters*. Number of flagellomeres in female and male: 9–10 (*Thoreauana*, Fig. 3[8]) / 10–11 (*Dilapothor*, Fig. 3[10]) / 11–11 (*Lytoxysta*, Fig. 3[6]) / 11–12 (all other). Shape of last two flagellomeres: wider than the rest and broadly jointed (*Apocharips*, *Dilapothor*, *Dilyta*, *Thoreauana*, Fig. 3[6–10]) / as wide as the previous with constriction between them (*Alloxysta*, *Lobopterocharips*, *Lytoxysta*, *Phaenoglyphis*, Fig. 3[1–6]). Pedicel: cup-shaped (*Lobopterocharips*, Fig. 3[5]) / cylindrical (all others).

*Specific characters*. Proportions (length and width) of pedicel, F1, F2, F3 and F4. Number of flagellomeres forming of club (in some species some flagellomeres are wider resembling a club). Number of flagellomeres with rhinaria. Males: F1, F2, F3 modified or not (curved, excavated, humped) (Fig. 3[2, 4]).

**Figure 4.**
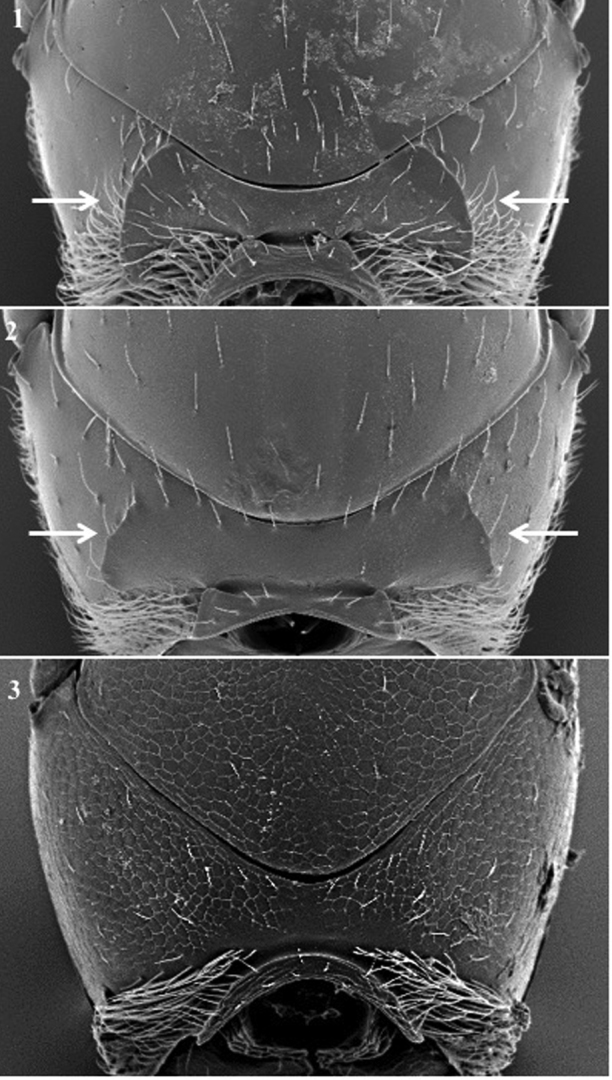
Pronotum. *Dilytasubclavata* (**1**); *Alloxystaxanthopsis* (**2**); *Lytoxystabrevipalpis* (**3**).

**Pronotum** (Fig. 4)

*Specific characters*. Lateral carinae: absent (Fig. 4[3]) / present (short (Fig. 4[2]) or long (Fig. 4[1]), reaching mesoscutum or not).

**Mesopleuron** (Fig. 1)

*Generic characters*. Mesopleural triangle absent (*Lytoxysta*, Fig. 1[3]) / present (all others). Mesopleural sulcus present (*Phaenoglyphis*, Fig. 1[2]) / absent (all others).

**Figure 5.**
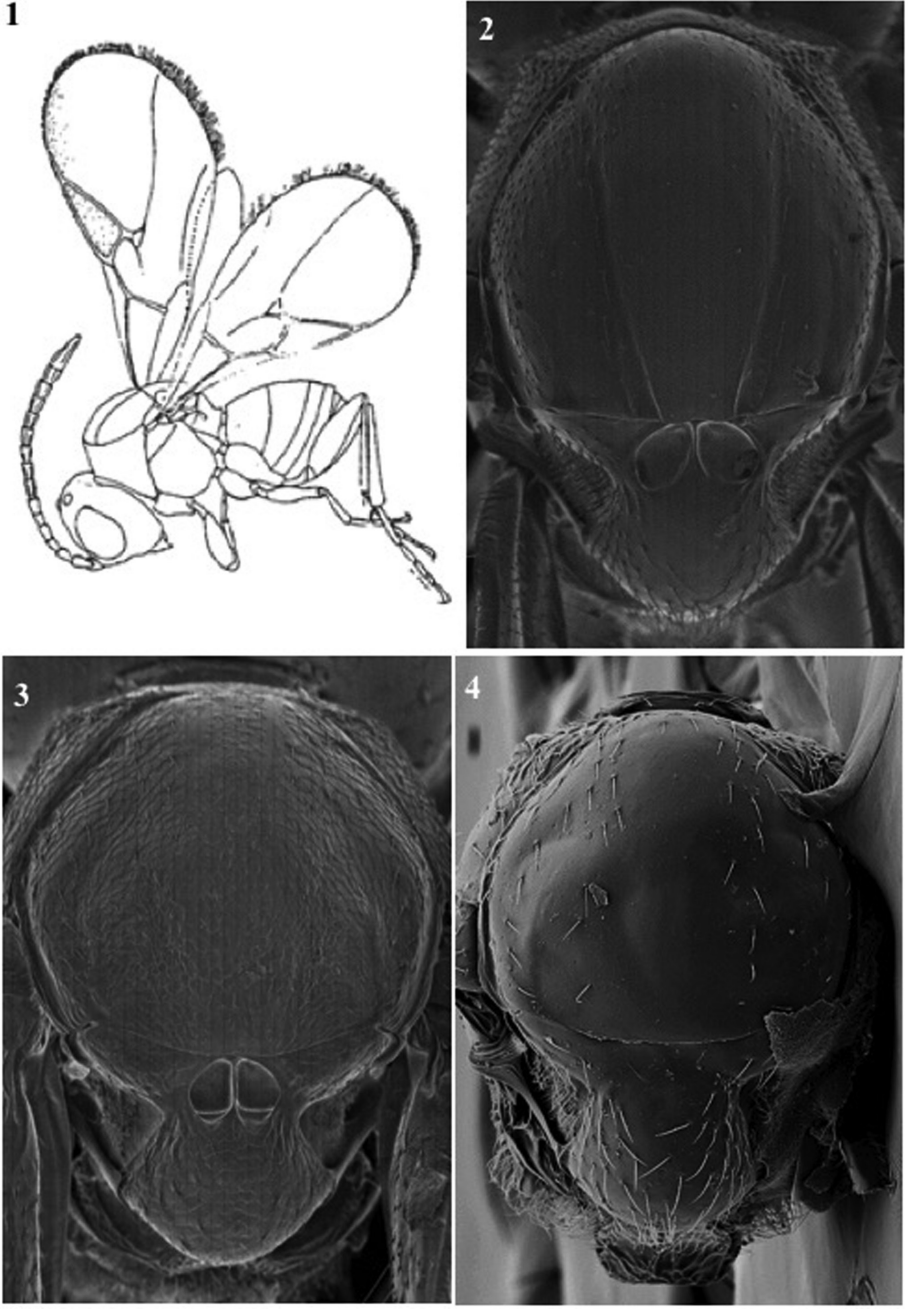
Mesoescutum. *Protocharipsevenhuisi* (**1**); *Phaenoglyphisinsperatus* (**2**); *Phaenoglyphisevenhuisi* (**3**); *Phaenoglyphisnigripes* (**4**).

**Mesoscutum** (Fig. 5)

*Generic characters*. Notauli present (only in †*Protocharips* and some *Phaenoglyphis*, Fig. 5[1,2]) / absent (all others).

*Specific characters*. Notauli present (Fig. 5[1, 2]) or absent (Fig. 5[4]) (only for *Phaenoglyphis* species). Presence or absence of very fine imbricate sculpture in basal areas of scutum (for *Phaenoglyphis* species, Fig. 5[3]).

**Figure 6.**
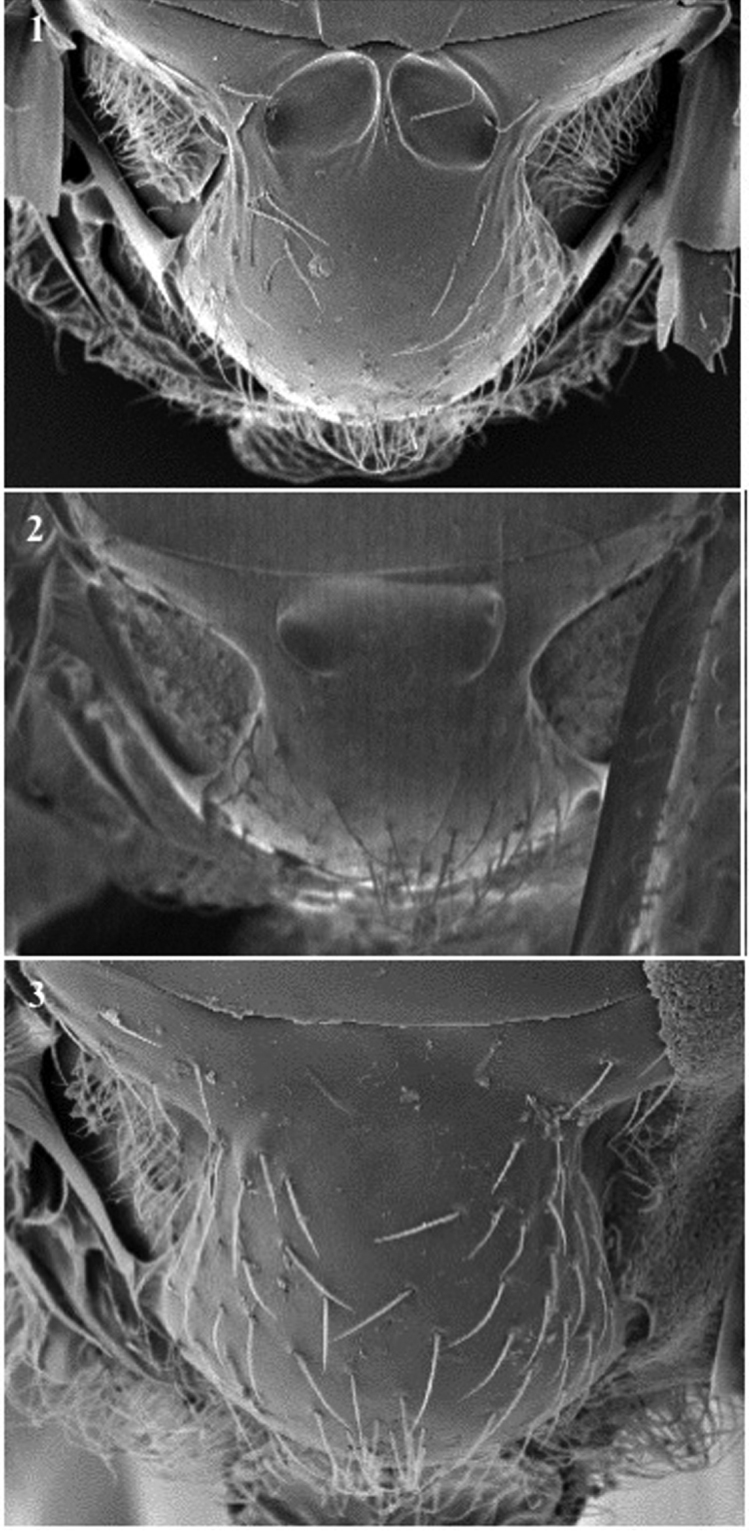
Scutellum. *Phaenoglyphisamericana* (**1**); *Phaenoglyphisvillosa* (**2**); *Phaenoglyphislaevis* (**3**).

**Figure 7.**
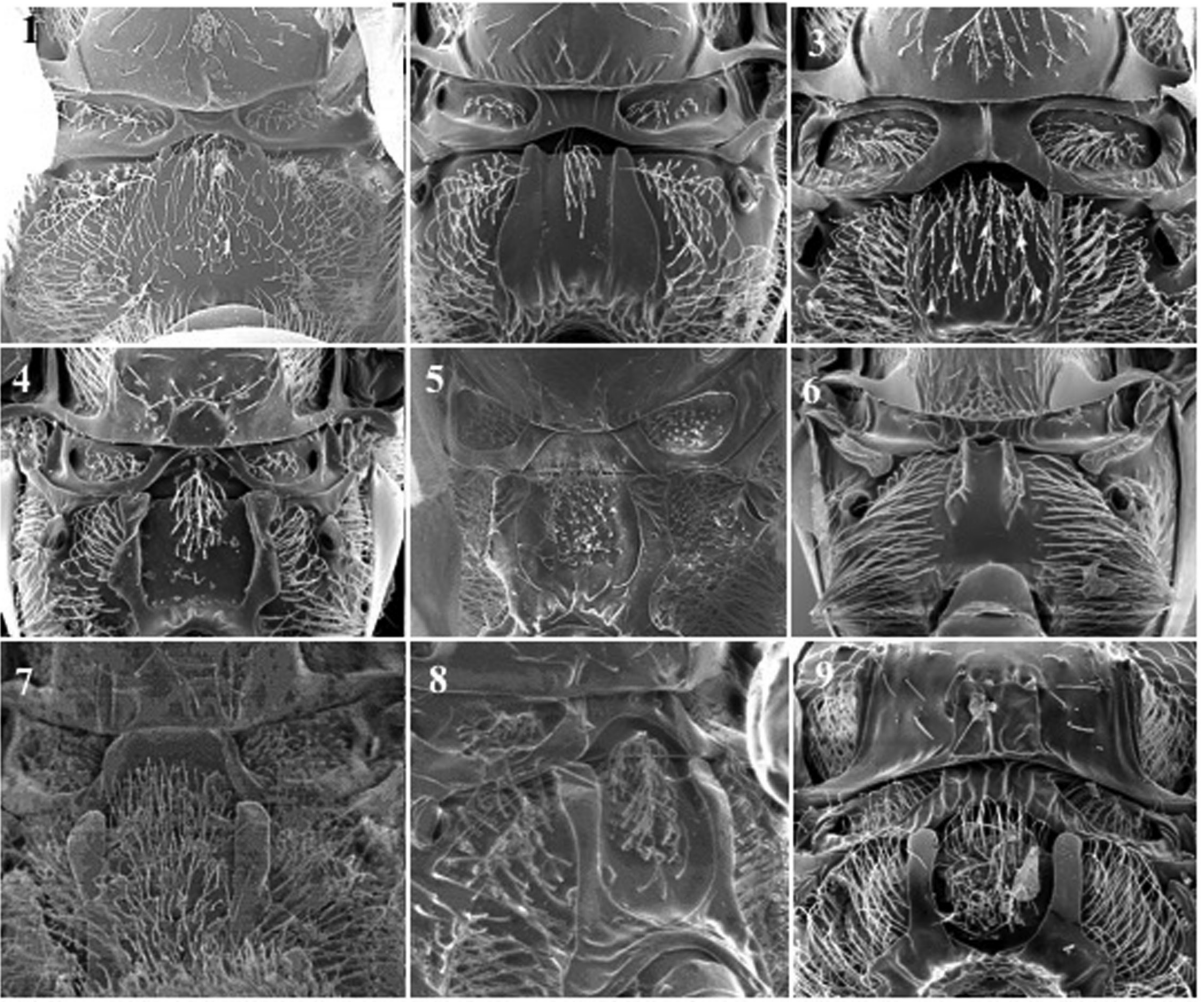
Scutellum and Propodeum. *Alloxystafuscicornis* (**1**); *Alloxystaxanthopsis* (**2**); *Phaenoglyphisamericana* (**3**); *Dilytasubclavata* (**4**); *Dilytaaustralafricana* (**5**); *Lytoxystabrevipalpis* (**6**); *Dilapothorcarverae* (**7**); *Thoreauanamascagnini* (**8**); *Apocharipstrapezoidea* (**9**).

**Scutellum** (Figs 6, 7)

*Generic characters*. Scutellar foveae present (only in some *Phaenoglyphis*, Fig. 6) / absent (all other). Posterodorsal extensions of axillar strip present (*Alloxysta*, *Lobopterocharips*, *Lytoxysta*, *Phaenoglyphis*, Fig. 10[1]) / absent (*Apocharips*, *Dilapothor*, *Dilyta*, *Thoreauana*, Fig. 10[2]). Carinae on scutellum apex: absent (*Phaenoglyphis*, *Lobopterocharips* and some *Alloxysta*, Newc) / longitudinal carinae at centre (some *Alloxysta*, Fig. 7[1]) / irregular carinae (*Lytoxysta*, Fig. 7[6]) / M-shaped carina at centre (*Apocharips*, Fig. 7[9]) / ∩-shaped carina or two long symmetrical carinae (*Dilyta*, Fig. 7[4, 5]) / two short symmetrical carinae (*Thoreauana*, Fig. 7[8]) / three small carinae at each side (*Dilapothor*, Fig. 7[7]).

*Specific characters*. Scutellar foveae absent (Fig. 6[3]) or present (fused, Fig. 6[2]; or unfused, Fig. 6[1, 2]) (for *Phaenoglyphis*). Carinae on scutellum apex: absent (Fig. 7[3]) or present (a single carina, Fig. 7[1], to several longitudinal carinae, Fig. 7[2]) (for *Alloxysta* species); two long symmetrical carinae (African *Dilyta*, Fig. 7[5]) or ∩-shaped carina (non-African *Dilyta*, Fig. 7[4]).

**Propodeum** (Fig. 7)

*Specific characters*. Presence or absence of longitudinal carinae; if present, shape of carinae (short, long, thin, broad, forming a plate). Pubescence.

**Figure 8.**
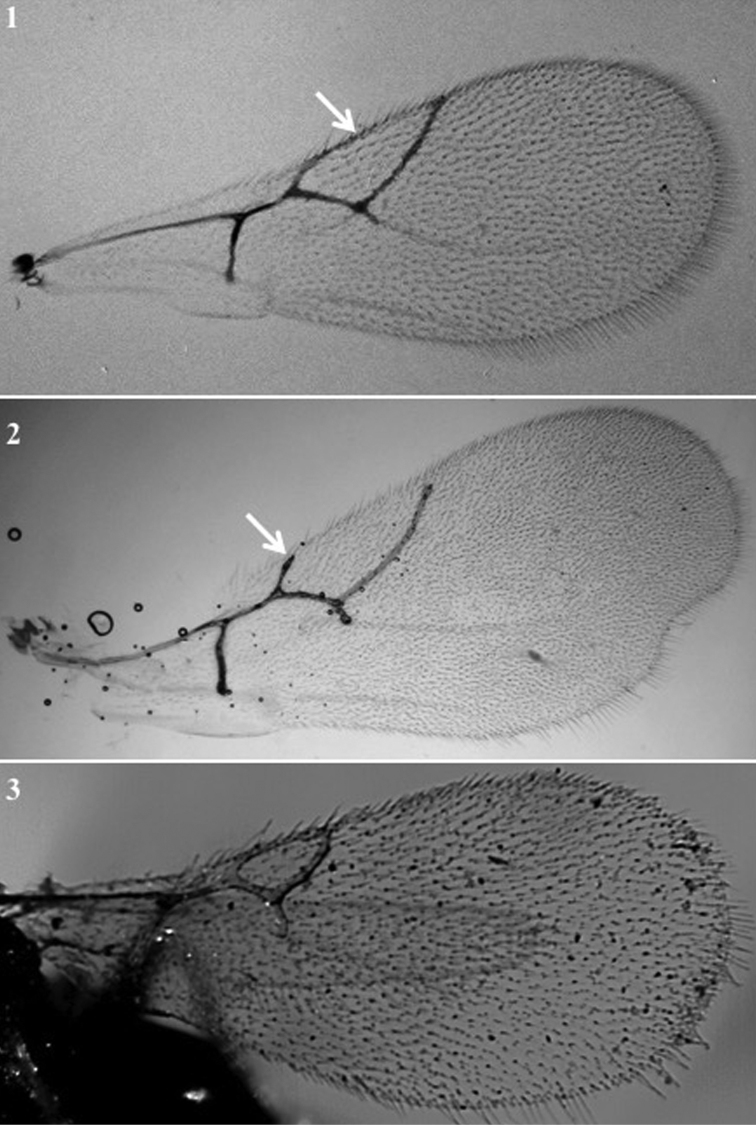
Forewing. *Phaenoglyphisvillosa* (**1**); *Lobopterocharipsarreplegata* (**2**); *Alloxystaruficollis* (**3**).

**Forewing** (Fig. 8)

*Generic characters*. Undulation in posteroapical margin of wing present (*Lobopterocharips*, Fig. 8[2]) / absent (all others, Fig. 8[1, 3]). Areola present (only in †*Protocharips*, Fig. 5[1]) / absent (all others).

*Specific characters*. Shape, size, and length of radial cell (Fig. 8).

**Figure 9.**
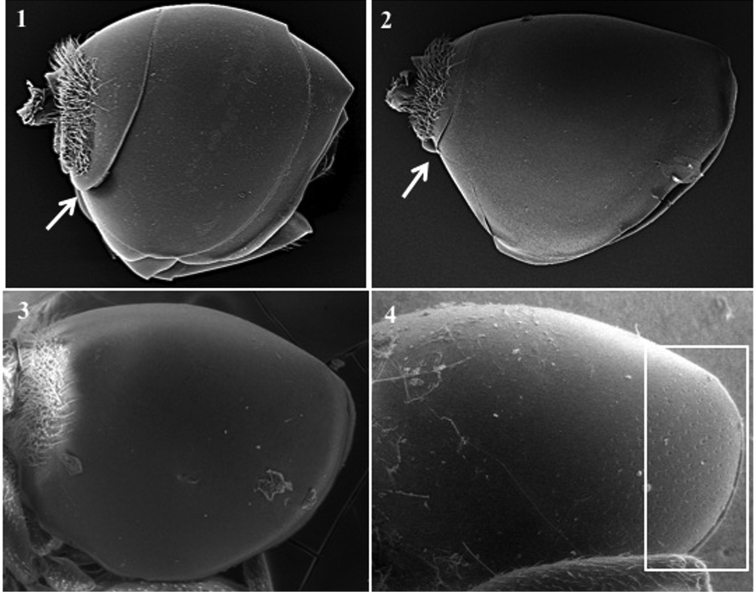
Metasoma. *Alloxysta* (**1**); *Apocharips* (**2**); *Dilyta*, without puntuation on distal area (**3**); *Dilyta*, with puntuation on distal area (**4**).

**Metasoma** (Fig. 9)

*Generic characters*. Metasoma with two visible large terga with subequal dorsomedial lengths (*Alloxysta*, *Lobopterocharips*, *Lytoxysta*, *Phaenoglyphis*, Fig. 9[1]; also in †*Protocharips*, Fig. 5[1]) / with a small basal tergum, terminating just posterior to ring of setae (*Apocharips*, Fig. 9[2]) / not segmented, only one tergite visible (*Dilapothor*, *Dilyta*, *Thoreauana*, Fig. 9[3, 4]).

*Specific characters*. Punctuation on distal area absent (Fig. 9[3]) or present (Fig. 9[4]) (*Dilyta* species).

**Figure 10.**
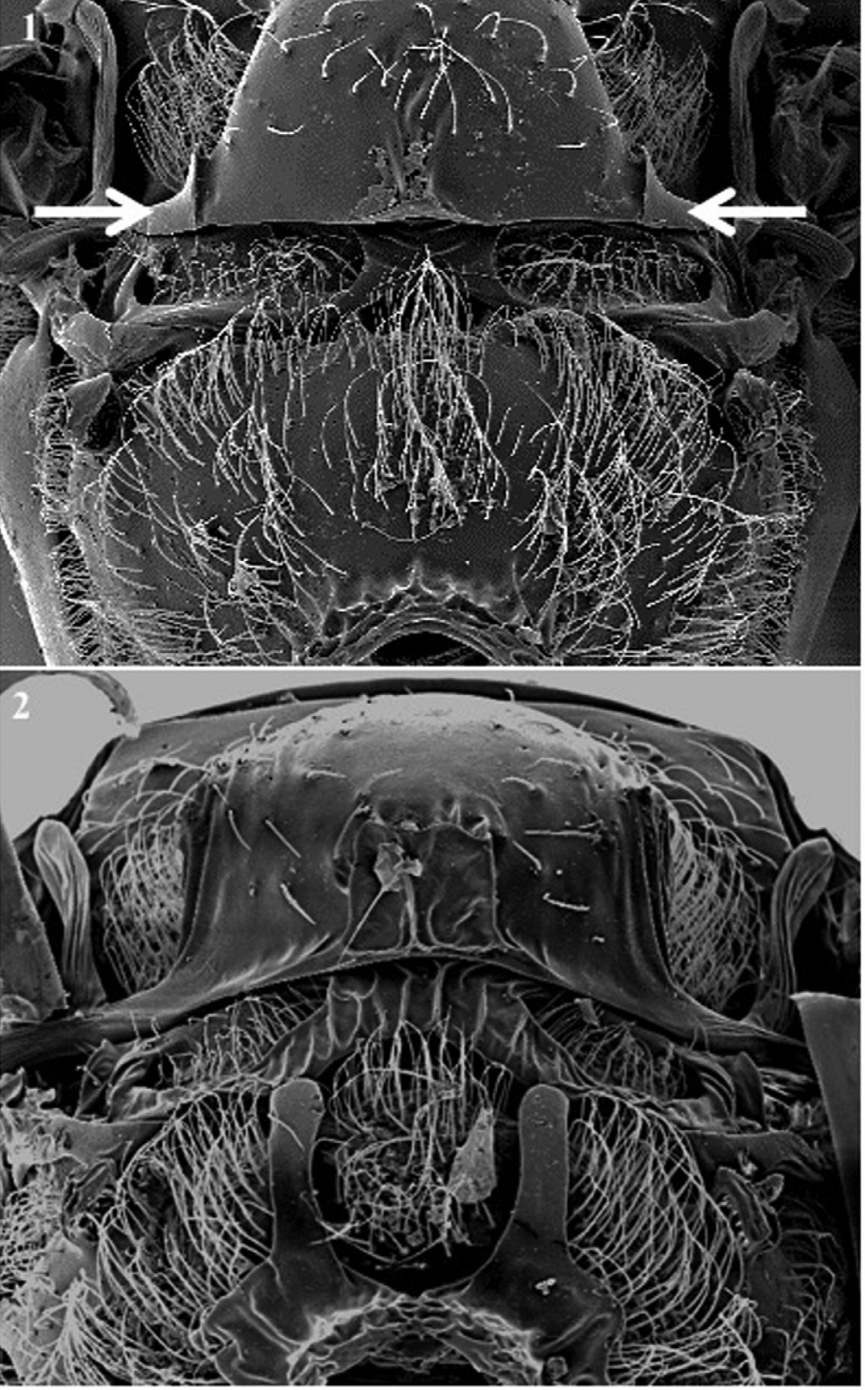
Mesosoma, posterior view. *Alloxystavictrix*, with axillar strip (indicated with arrows) (**1**); *Apocharipstrapezoidea*, without axillar strip (**2**).

### Key to genera

**Table:** 

1	Metasoma with two large terga visible, subequal in length along middorsal line, but basal tergite 1/4–1/3 smaller than second terga in lateral view (Fig. 11[1]). Antenna with all flagellomeres separated by constrictions (Fig. 11[8]). Posterodorsal extensions of axillar strip present (Fig. 11[11]). Hyperparasitoids of Aphididae (for those genera where it is known)	**2**
–	Metasoma with a single tergal plate, or if two, then basal tergite much shorter than second along middorsal line (Fig. 11[2, 3]). Antenna with last two flagellomeres broadly jointed or fused (Fig. 11[7]). Posterodorsal extension of axillar strip absent (Fig. 11[12]). Hyperparasitoids of Psyllidae (for those genera where it is known)	**5**
2	Mesopleuron with horizontal sulcus in lower part (Fig. 11[4])	***Phaenoglyphis* Förster, 1869**
–	Mesopleuron without horizontal sulcus (Fig. 11[5])	**3**
3	Forewing with an undulation in the apical part of the posterior margin (Fig. 11[17]). Antenna with a cup-shaped pedicel (Fig. 11[10]). Known only from Nepal	***Lobopterocharips* Paretas-Martínez & Pujade-Villar, 2007**
–	Margin of the forewing continuous (Fig. 11[16]). Antenna with a cylindrical pedicel (Fig. 11[9])	**4**
4	Mesopleuron without mesopleural triangle (Fig. 11[6]). Head and mesosoma with fine reticulate sculpture. Nearctic	***Lytoxysta* Kieffer, 1909**
–	Mesopleuron with mesopleural triangle (Fig. 11[4, 5]). Head and mesosoma smooth, unsculptured. Cosmopolitan	***Alloxysta* Förster, 1869**
5	Metasoma with a small basal tergum, terminating just posterior to ring of setae (Fig. 11[2]). R1 long, reaching wing margin. Palaearctic and Neotropical	***Apocharips* Fergusson, 1986**
–	Metasoma appearing unsegmented, only one tergite visible (Fig. 11[3]). R1 short, not reaching wing margin	**6**
6	Apex of scutellum with a ∩-shaped projected plate (Fig. 11[12]) or with one carina on each side, both symmetrical and parallel higher than axillar strip; distance between them equal to distance between propodeal carinae (Fig. 11[13]). Female antenna with 11 flagellomeres. Cosmopolitan except Neotropics and Australia	***Dilyta* Förster, 1869**
–	Apex of scutellum without projected plate, with symmetrical carinae longer than axillar strip (Fig. 11[13–15]). Female antenna with less than 11 flagellomeres	**7**
7	Head higher than broad in anterior view. Female antenna with 10 flagellomeres, apical club two-segmented. Three carinae at each side of the scutellum apex (Fig. 11[14]). Radial cell large, 2r as long as Sc+R1; Rs long and curved and giving an elongated aspect to the radial cell. Australia	***Dilapothor* Paretas-Martínez & Pujade-Villar, 2006**
–	Head rounded in anterior view. Female antenna with 9 flagellomeres, apical club not segmented (Fig. 11[7]). One small carina presents at each side of the scutellum apex. (Fig. 11[15]). Radial cell small, 2r shorter than Sc+R1; Rs short and almost straight. Australia	***Thoreauana* Girault, 1930**

**Figure 11.**
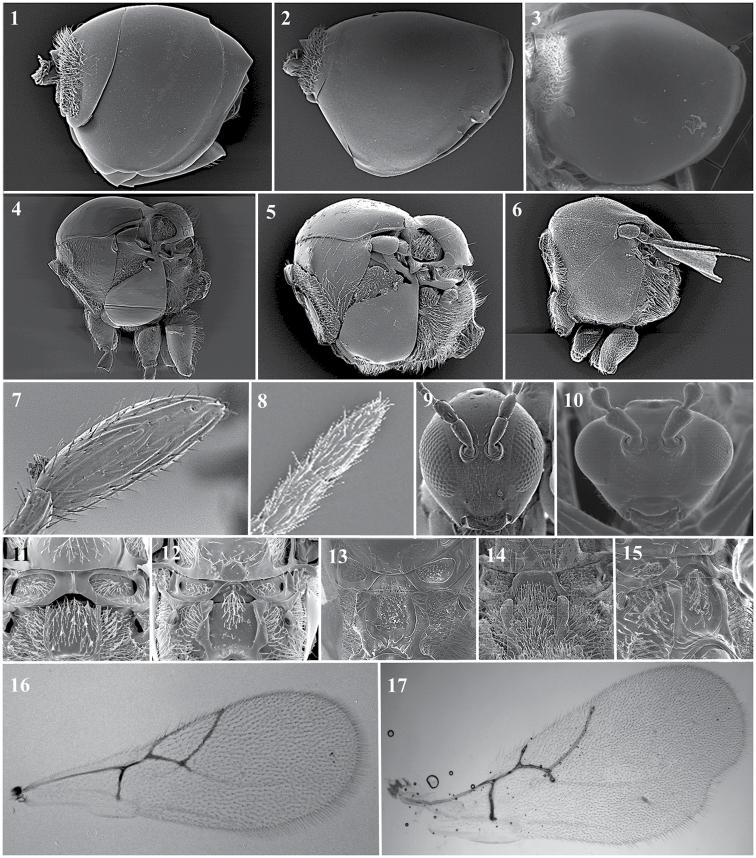
Charipinae general features. metasoma *Alloxysta* sp. (**1**); metasoma *Apocharips* sp. (**2**); metasoma *Dilyta* sp. (**3**); mesosoma *Phaenoglyphis* sp. (**4**); mesosoma *Alloxysta* sp. (**5**); mesosoma *Lytoxysta* sp. (**6**); two last flagellomeres *Thoreauana* sp. (**7**); two last flagellomeres *Phaenoglyphis* sp. (**8**); head *Thoreauana* sp. (**9**) ; head *Lobopterocharips* sp. (**10**); propodeum *Phaenoglyphis* sp. (**11**); propodeum holarctic *Dilyta* sp. (**12**); propodeum african *Dilyta* sp. (**13**); propodeum *Dilapothor* sp. (**14**); propodeum *Thoreauana* sp. (**15**); fore wing *Phaenoglyphis* sp. (**16**); fore wing *Lobopterocharips* sp. (**17**).

#### Alloxysta

Taxon classificationAnimaliaHymenopteraFigitidae

Förster, 1869

Allotria Westwood, 1833: 494. Type: Allotriavictrix Westwood, 1833. Homonym of Allotria Hübner, 1823: 280. Synonymized by Hellén (1963: 8).
Xystus Hartig, 1840: 199. Type: Xystuserythrocephalus Hartig, 1840. Homonym of Xystus Schoenherr, 1826: 310. Synonymized by Hellén (1963: 8).
Alloxysta Förster, 1869: 338. Type: Xystusmacrophadnus Hartig, 1841.
Pezophycta Förster, 1869: 338. Type: Xystusbrachypterus Hartig, 1840. Synonymized by Hellén (1963: 8).
Nephycta Förster, 1869: 338. Type: Nephyctadiscreta Förster, 1869. Synonymized by Hellén (1963: 8).
Adelixysta Kierych, 1988: 351. Type: Adelixystasawoniewiczi Kierych, 1988. Synonymized by Menke and Evenhuis (1991: 150).
Carvercharips Kovalev, 1994: 413, 414. Type: Alloxystacarinata Carver, 1992. Synonymized by Paretas-Martínez et al. (2007: 161).

##### General features.

*Head.* Transversally ovate, smooth and shiny, slightly wider than high in anterior view.

**Figure 12.**
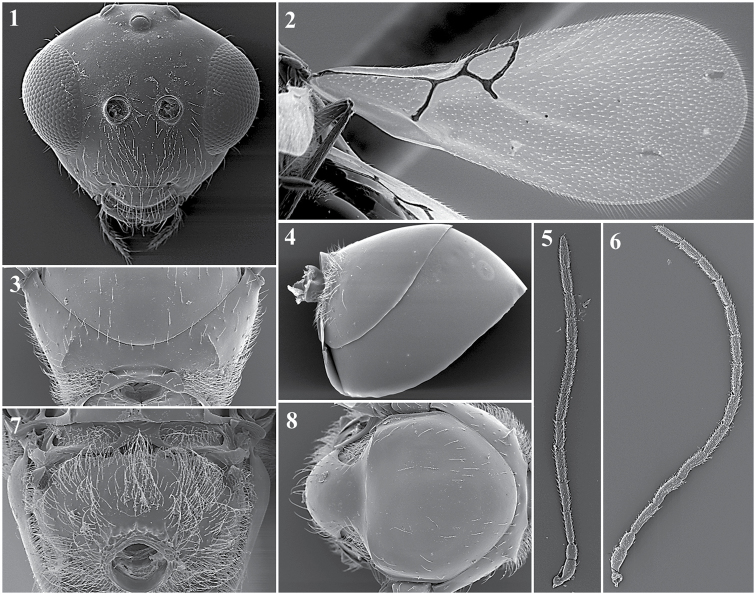
*Alloxysta* general features. Head (**1**); fore wing (**2**); pronotum (**3**); metasoma (**4**); female antennae (**5**); male antennae (**6**); propodeum (**7**); mesoscutum (**8**).

Setae found below, between and above toruli, on vertex and multiple setae on the face. Transfacial distance is 0.9–1.3× the height of the compound eye. Malar space is 0.3–0.6× the height of the compound eye (Fig. 12[1]).

*Antenna.* Female: 13-segmented, filiform. All antennomers covered with sparse setae (Fig. 12[5]). Male: 14-segmented, filiform. All antennomers covered with sparse setae (Fig. 12[6]).

*Mesosoma.* Pronotum with scattered setae that are differently distributed, depending on the species, with or without carinae (Fig. 12[3]). Mesoscutum is smooth and shiny, round in the dorsal view, with sparse setae. Scutellum is smooth and shiny with scattered setae that are usually more abundant on the apex (Fig. 12[8]). Propodeum with multiple setae, with or without carinae; carinae are separated or fused, forming a variably shaped plate (Fig. 12[7]).

*Forewing*. Longer than the body, 1.4–1.8× as long as the mesosoma and metasoma together, with dense pubescence; marginal setae present (Fig. 12[2]).

*Metasoma.* Anterior region has an incomplete ring of setae, is glabrous at centre and is wider laterally. Metasoma is smooth and shiny, with T3 and T4 clearly separated (Fig. 12[4]).

##### Distribution.

Cosmopolitan (Ferrer-Suay et al. 2012)

##### Hosts.

Endoparasitoids of Aphidiinae (Hymenoptera, Braconidae) and Aphelininae (Hymenoptera, Braconidae) that are endoparasitoids of aphids (Hemiptera, Aphididae) (Fergusson 1986; Menke and Evenhuis 1991). Found in a variety of hosts (Ferrer-Suay et al. 2012).

### Key to species

**Table:** 

1	Brachypterous species	**2**
–	Fully winged species, usually longer than mesosoma+metasoma	**9**
2	Forewing reaching the end of metasoma; visible radial cell	**3**
–	Forewing reaching the beginning of the metasoma or shorter; without radial cell visible	**6**
3	Radial cell completely open (Fig. 18[8])	***A.marshalliana* (Kieffer, 1900)**
–	Radial cell closed	**4**
4	Pronotal carinae absent	***A.glebaria* (Hellén, 1963)**
–	Pronotal carinae present	**5**
5	Propodeal carinae absent	***A.pseudofuscicornis* (Ferrer-Suay, 2017)**
–	Propodeal carinae present	***A.curta* (Ferrer-Suay, 2017)**
6	Pronotal carinae present; propodeal carinae absent. Females sometimes brachypterous. Fully winged female has a closed radial cell, 2.4× as long as wide	***A.halterata* (Thomson, 1862)**
–	Pronotal carinae absent; propodeal carinae absent or present. When present, female always brachypterous	**7**
7	Propodeal carinae present; F1 shorter than pedicel (Fig. 13[13])	***A.brachyptera* (Hartig, 1840)**
–	Propodeal carinae absent. F1 shorter or longer than pedicel	**8**
8	Forewing reaches the beginning of the metasoma. Female: F1 longer than pedicel (Fig. 15[6]). Male: F1 subequal or slightly longer than pedicel, F1–F4 subequal in length	***A.pedestris* (Curtis, 1838)**
–	Forewing short, practically absent. Female: F1 shorter than pedicel (Fig. 13[6]). Male: unknown	***A.apteroidea* (Hellén, 1963)**
9	Radial cell completely or partially open	**10**
–	Radial cell closed	**69**
10	Radial cell completely open	**11**
–	Radial cell partially open	**41**
11	Propodeal carinae absent	**12**
–	Propodeal carinae present	**24**
12	Pronotal carinae absent	**13**
–	Pronotal carinae present	**14**
13	F2 longer than F1 and F3 (Fig. 15[12]); radial cell 2.3× as long as wide (Fig. 18[28])	***A.proxima* (Belizin, 1962)**
–	F2 shorter than F1 and subequal to F3 (Fig. 16[5]); radial cell 2.7× as long as wide (Fig. 19[23])	***A.huberi* (Ferrer-Suay & Pujade-Villar, 2014)**
14	F2 longer than F1 and F3 (Fig. 16[1]); radial cell 3.8× as long as wide (Fig. 19[19])	***A.alpina* (Ferrer-Suay & Pujade-Villar, 2014)**
–	F2 shorter than or subequal to F1 and F3; radial cell shorter	**15**
15	Female: unknown. Male: rhinaria and club shape begin at F1; F1 and F2 curved, F1 longer than pedicel and F2, F2–F4 nearly equal in length (Fig. 15[34]); radial cell 4.1× as long as wide (Fig. 19[18])	***A.centroamericana* (Ferrer-Suay & Pujade-Villar, 2013)**
–	Female and/or male: rhinaria and club shape begin in different flagellomeres; flagellomere differently proportioned; radial cell shorter	**16**
16	Female: unknown. Male: rhinaria and club shape begin at F2; F2 and F3 are curved; F1 shorter than or subequal to F2, F2 subequal to F3, F3 longer than F4 (Fig. 15[29]); radial cell 2.8× as long as wide (Fig. 19[13])	***A.vandenboschi* (Andrews, 1978)**
–	Female and/or male: rhinaria and club shape begin at F3 or F4; flagellomere differently proportioned; radial cell not equal to 2.8× as long as wide	**17**
17	Female: F1–F4 subequal in length (Fig. 14[16]). Male: F1 with a lateral hump; radial cell 2.7× as long as wide (Fig. 18[7])	***A.mara* (Paretas-Martínez & Pujade-Villar, 2005)**
–	Female: F1–F4 subequal in length. Male: F1 without a lateral hump; radial cell longer or shorter, not equal to 2.7× as long as wide	**18**
18	Female: F2 subequal to F1, F2 longer than F3 (Fig. 13[11]); radial cell 3.0× as long as wide (Fig. 17[10]). Male: unknown	***A.basimacula* (Cameron, 1886)**
–	Female: F2 shorter than F1, F2 can be longer, shorter than or subequal to F3; radial cell longer or shorter, not equal to 3.0× as long as wide	**19**
19	Body is covered in abundant pubescence; radial cell 4.9× as long as wide in females (Fig. 18[25]) and 3.2× as long as wide in males	***A.pilosa* (Ferrer-Suay & Pujade-Villar, 2013)**
–	Body is covered in scattered setae; radial cell < 4.9× as long as wide in females and < 3.2× as long in males	**20**
20	F2 longer than F3 (Fig. 13[12]); radial cell 2.7× as long as wide (Fig. 17[11])	***A.brachycera* (Hellén, 1963)**
–	F2 shorter than or subequal to F3; radial cell shorter or longer than, not equal to 2.7× as long as wide	**21**
21	F2–F4 subequal in length (Fig. 13[23]); radial cell 2.8× as long as wide (Fig. 17[21])	***A.crassa* (Cameron, 1889)**
–	F2–F4 unequal in length; radial cell < 2.8× as long as wide	**22**
22	F2 subequal to F3 (Fig. 14[26]); radial cell 2.3× as long as wide (Fig. 18[17])	***A.nipona* (Ferrer-Suay & Pujade-Villar, 2013)**
–	F2 shorter than F3; radial cell more than 2.3× as long as wide	**23**
23	F1 4.4× as long as wide, F3 longer than F4 (Fig. 14[25]); radial cell 2.9× as long as wide, Rs and R1 reach the costal margin (Fig. 17[16])	***A.nigrita* (Thomson, 1862)**
–	F1 1.4× as long as wide, F3 subequal to F4 (Fig. 15[7]); radial cell 2.8× as long as wide, Rs and R1 do not reach the costal margin (Fig. 18[23])	***A.piceomaculata* (Cameron, 1883)**
24	Two propodeal carinae well defined, independently reaching the base	**25**
–	Propodeum with two carinae, which form a plate	**27**
25	Female: rhinaria and club shape begin at F3; F1 longer than pedicel and F2, F2 shorter than F3, F3 subequal to F4 (Fig. 15[4]); few carinae on apex of scutellum; radial cell 2.4× as long as wide (Fig. 18[21]). Male: unknown	***A.paretasmartinezi* (Ferrer-Suay & Pujade-Villar, 2013)**
–	Rhinaria and club shape begin in other flagellomeres; thick, parallel carinae on the apex of scutellum; radial cell shorter or longer than, not equal to 2.4× as long as wide	**26**
26	Female: rhinaria and club shape begin at F5; F2 subequal to F3 (Fig. 13[16]); radial cell 2.2× as long as wide (Fig. 17[14])	***A.carinata* (Carver, 1992)**
–	emale: rhinaria and club shape begin at F4; F2 shorter than F3 (Fig. 15[20]); radial cell 2.8× as long as wide (Fig. 19[4])	***A.samurai* (Ferrer-Suay & Paretas-Martínez, 2013)**
27	Rhinaria and club shape begin at F2	**28**
–	Rhinaria and club shape begin in other flagellomere	**29**
28	Female: F2–F4 subequal in length (Fig. 15[3]). Male: F2 slightly curved and longer than F3; propodeum with two well defined carinae and separated in the first half with setae present, joining to form a plate in the last half; radial cell 2.6× as long as wide (Fig. 18[20])	***A.pallidicornis* (Curtis, 1838)**
–	Female: F2 longer than F3, F3 longer than F4 (Fig. 13[1]). Male: F2 shorter than F3; propodeum with two carinae joining to form a thick plate; setae on top and curved sides; radial cell 2.2× as long as wide (Fig. 17[1])	***A.abdera* (Fergusson, 1986)**
29	Female: unknown. Male: rhinaria and club shape begin at F1; F1 curved; F1–F4 subequal in length (Fig. 16[10]); radial cell 3.3× as long as wide (Fig. 19[28])	***A.vicenti* (Ferrer-Suay, 2014)**
–	Rhinaria and club shape begin in other flagellomeres; different flagellomere proportions; various sizes of radial cells	**30**
30	Rhinaria and club shape begin at F3	**31**
–	Rhinaria and club shape begin at F4	**33**
31	F1 subequal to pedicel, F2 subequal to F3, F3 shorter than F4 (Fig. 16[15]); radial cell 2.4× as long as wide (Fig. 19[33]). Male: unknown	***A.nottoni* (Ferrer-Suay & Pujade-Villar, 2015)**
–	F1 longer than pedicel	**32**
32	Female: F1 2.9× as long as wide, F2 shorter than F3, F3 subequal to F4 (Fig. 15[32]); radial cell 2.4× as long as wide (Fig. 19[16]). Male: unknown	***A.xanthopa* (Thomson, 1862)**
–	A different combination of features	**33**
33	Pronotal carinae absent	**34**
–	Pronotal carinae present	**35**
34	Rhinaria and club shape being at F3 (Fig. 14[18]). Male: F1 is 3.9× as long as wide; F2 subequal to F3, F3 shorter than F4; radial cell 2.5× as long as wide in females and 2.3× as long in males (Fig. 18[9])	***A.medinae* (Ferrer-Suay & Pujade-Villar, 2012)**
–	Rhinaria and club shape being at F4; radial cell 2.4× as long as wide (Fig. 19[22])	***A.franca* (Ferrer-Suay & Pujade-Villar, 2014)**
35	F1 shorter than or subequal to pedicel	**36**
–	F1 longer than pedicel	**39**
36	F1 shorter than pedicel	**37**
–	F1 subequal to pedicel	**38**
37	F2 slightly longer than F1 and F3 (Fig. 16[9]); radial cell 2.5× as long as wide (Fig. 19[27])	***A.texanae* (Ferrer-Suay & Pujade-Villar, 2014)**
–	F2 shorter than F1 and subequal to F3 (Fig. 16[14]); radial cell 2.0× as long as wide (Fig. 19[32])	***A.pascuali* (Ferrer-Suay, 2018)**
38	F2 shorter than F1 and F3 (Fig. 16[2]); radial cell 3.8× as long as wide (Fig. 19[20]); irregular, rounded carina on apex of scutellum	***A.areeluckator* (Ferrer-Suay & Pujade-Villar, 2014)**
–	F2 subequal to F1 and longer than F3 (Fig. 16[13]); radial cell 2.3× as long as wide (Fig. 19[31]); no carina on apex of scutellum	***A.palearctica* (Ferrer-Suay & Pujade-Villar, 2018)**
39	Pronotal carinae thick, long and clearly visible; propodeum with two carinae joining to form a width plate; radial cell 2.2× as long as wide (Fig. 19[25])	***A.pili* (Ferrer-Suay, 2014)**
–	Pronotal and propodeal carinae different; radial cell > 2.2× as long as wide	**40**
40	Pronotum with two thick and short carinae covered by few setae; propodeum with two straight and parallel carinae joining at the base and covered by abundant pubescence; radial cell 3.2× as long as wide (Fig. 17[19])	***A.costaricensis* (Ferrer-Suay & Pujade-Villar, 2011)**
–	Pronotum with two thick and long carinae, rounded, curving and clearly visible; propodeum with two straight carinae that are well defined on top, forming a plate in the last half, with strongly curved sides; radial cell 2.3× as long as wide (Fig. 19[10])	***A.thorpei* (Ferrer-Suay & Pujade-Villar, 2012)**
41	Propodeal carinae absent or only slightly defined on top	**42**
–	Propodeal carinae present	**54**
42	Pronotal carinae absent	**43**
–	Pronotal carinae present	**46**
43	F1 longer than pedicel	**44**
–	F1 subequal to pedicel	**45**
44	Setae absent where the carinae are usually present; F1 5.0× as long as wide (Fig. 16[3]); radial cell 2.8× as long as wide (Fig. 19[21])	***A.buffingtoni* (Ferrer-Suay & Pujade-Villar, 2014)**
–	Propodeum completely covered by setae; F1 is 3.0× as long as wide (Fig. 15[25]); radial cell 2.3× as long as wide (Fig. 19[9])	***A.soluta* (Hellén, 1963)**
45	F1 longer than F2, F2 subequal to F3 (Fig. 15[5]); radial cell 2.8× as long as wide; Rs vein does not reach the costal margin (Fig. 18[22])	***A.patens* (Hellén, 1963)**
–	F1–F3 subequal in length (Fig. 14[13]); radial cell 2.3× as long as wide; Rs vein reaches the costal margin (Fig. 18[4])	***A.longiventris* (Baker, 1896)**
46	Thick carinae on apex of scutellum; F1 longer than pedicel and F2, F2–F4 subequal (Fig. 15[16])	***A.rubidus* (Ferrer-Suay & Pujade-Villar, 2012)**
–	Carinae absent on apex of scutellum; different flagellomere proportions	**47**
47	F1 subequal to pedicel (Fig. 14[21]). Male: F2 and F3 curved. Radial cell 2.5× as long as wide (Fig. 18[12])	***A.minuscula* (Andrews, 1978)**
–	F1 longer than pedicel; various sizes of radial cells	**48**
48	F2–F4 subequal in length	**49**
–	F2–F4 unequal in length	**52**
49	Rhinaria and club shape begin at F4; F1 longer or slightly shorter than F2 (Fig. 14[2])	***A.fuscipes* (Thomson, 1862)**
–	Rhinaria and club shape begin in other flagellomere	**50**
50	Rhinaria and club shape begin at F1 (Fig. 15[23]); radial cell 2.9× as long as wide (Fig. 19[7]). Male: unknown	***A.sharkeyi* (Ferrer-Suay & Pujade-Villar, 2013)**
–	Rhinaria and club shape begin at F3; radial cell longer or shorter, not equal to 2.9× as long as wide	**51**
51	Female: F1 is 6.3× as long as wide, F2 4.6× as long as wide (Fig. 15[18]); radial cell 2.6× as long as wide (Fig. 19[2]). Male: unknown	***A.salicicola* (Belizin, 1973)**
–	Female: F1 is 3.5× as long as wide, F2 is 2.1× as long as wide (Fig. 15[22]); radial cell 2.7× as long as wide (Fig. 19[6]). Male: rhinaria and club shape begin at F4; F1 longer than F2, F2 longer than F3, F3 shorter than F4	***A.semiaperta* (Fergusson, 1986)**
52	Female: rhinaria and club shape begin at F4 (Fig. 13[20]). Male: rhinaria and club shape begin at F2; none of the flagellomere are curved; F1 longer than pedicel, F1–F3 subequal, F3 shorter than or subequal to F4; radial cell 2.2× as long as wide (Fig. 17[18])	***A.commensuratus* (Andrews, 1978)**
–	Female and/or male: rhinaria and club shape begin at F3; flagellomere differently proportioned; size of radial cells diverse	**53**
53	Female: F1 subequal to F2, F2 longer than F3, F3 subequal to F4 (Fig. 14[15]). Male: F2 and F3 curved; F1 subequal to F2, F2 longer than F3, F3 longer than F4; large radial cell 3.0× as long as wide in both males and females (Fig. 18[6])	***A.macrophadna* (Hartig, 1841)**
–	Female: F1 longer than F2, F2 shorter than or subequal to F3, F3 shorter than F4 (Fig. 15[2]). Male: flagellomeres not curved; F1 longer than F2, F2 longer than F3, F3 shorter than F4; radial cell < 3.0× as long as wide in both sexes (Fig. 18[19])	**54**
54	Radial cell 2.5× as long as wide; Rs-2 strongly curved and radial cell wide open	***A.simplex* (Watanabe, 1950)**
–	Radial cell 2.7× as long as wide; Rs-2 not strongly curved and radial cell not wide open	***A.obscurata* (Hartig, 1840)**
55	Propodeal carinae do not protrude; F1 subequal to pedicel in both sexes; rhinaria and club shape begin at F4 (Fig. 13[19]); radial cell 2.1× as long as wide (Fig. 17[17])	***A.citripes* (Thomson, 1862)**
–	Propodeal carinae well defined and protruding; without the combination of characters as above	**56**
56	Propodeum with two well-defined carinae, which independently reach the base; carinae thick with curved sides; rhinaria and club shape begin at F3 in female (Fig. 15[10]); F1–F3 slightly curved in male; radial cell is small with a straight Rs vein (Fig. 18[26])	***A.pleuralis* (Cameron, 1879)**
–	Propodeum with two carinae, which form a plate or join together only at the base; without the combination of characters as above	**57**
57	Propodeal carinae thick, well defined, with curved sides joined at the base. Female: F1 subequal to pedicel, F1 longer than F2, F2 shorter than F3, F3 shorter than F4 (Fig. 13[8]). Male: F1 shorter than pedicel, F2–F4 subequal	***A.asiatica* (Ferrer-Suay & Pujade-Villar, 2013)**
–	Propodeal carinae joined at the base, forming a complete plate; male and female flagellomeres not proportioned as above	**58**
58	Rhinaria begin at F3 and club shape begins at F2; F3 subequal to pedicel and shorter than F4 (Fig. 14[14]); apex of scutellum has thick carinae	***A.luismii* (Ferrer-Suay, 2011)**
–	Rhinaria and club shape begin in the same flagellomere; F3 unequal to pedicel; no apex of scutellum without carinae	**59**
59	Female: unknown. Male: rhinaria and club shape begin at F2; F1 longer than F2, F2–F4 subequal; F2 slightly curved (Fig. 16[11]); radial cell 2.8× as long as wide (Fig. 19[29])	***A.viellae* (Ferrer-Suay & Pujade-Villar, 2013**)
–	Rhinaria and club shape begin in other flagellomeres; variety of combinations of the features explained above	**60**
60	Rhinaria and club shape begin at F4	**61**
–	Rhinaria and club shape begin at F3	**67**
61	Pronotal carinae absent	**62**
–	Pronotal carinae present	**64**
62	Female: F3 subequal to pedicel (Fig. 15[17]); propodeum with two carinae that form a plate with straight sides	***A.rufiventris* (Hartig, 1840)**
–	Female: F3 unequal to pedicel; propodeum with two carinae that form a plate with curved sides	**63**
63	Female: F1 longer than pedicel (Fig. 13[4]); propodeum with two carinae that form a plate with curved sides; radial cell 2.1× as long as wide (Fig. 17[4])	***A.antsirananae* (Ferrer-Suay & Pujade-Villar, 2012)**
–	Female: F1 shorter than pedicel (Fig. 15[24]); propodeum with two carinae that form a plate with only slightly-curved sides; radial cell 2.2× as long as wide (Fig. 19[8])	***A.slovenica* (Ferrer-Suay & Pujade-Villar, 2013)**
64	F1 subequal to pedicel	**65**
–	F1 longer than pedicel	**66**
65	Body brown; pronotum with scattered setae; found in *Aphis* sp.	***A.postica* (Hartig, 1841)**
–	Body bicolored; pronotum with abundant setae; found in *Neuquenaphis* sp.	***A.nothofagi* (Andrews, 1976)**
66	Female: F2 subequal to F3 (Fig. 13[17]); radial cell 2.3× as long as wide (sometimes the club shape begins at F3) (Fig. 17[15])	***A.castanea* (Hartig, 1841)**
–	Female: F2 shorter than F3 (Fig. 13[9]); radial cell 3.0× as long as wide (Fig. 17[8]). Male: unknown	***A.aurata* (Belizin, 1968)**
67	Female: F3 subequal to pedicel (Fig. 14[20]). Male: F1 longer than pedicel and F2, F2 subequal to F3; radial cell 2.0× as long as wide (Fig. 18[11])	***A.melanogaster* (Hartig, 1840)**
–	F1 longer than pedicel and F2; without combination of characters as above	**68**
68	Apex of scutellum without carina present; plate propodeum with straight sides	***A.longipennis* (Hartig, 1841)**
–	Apex of scutellum with a thick carina present; plate propodeum with curved sides and few setae on top	***A.andrewsi* (Ferrer-Suay & Pujade-Villar, 2011)**
69	Propodeal carinae present	**70**
–	Propodeal carinae absent	**91**
70	Propodeal carinae independent, slightly fused at bottom	***A.barbotini* (Ferrer-Suay & Pujade-Villar, 2016)**
–	Propodeal carinae fused, forming a plate	**71**
71	Pronotal carinae present	**72**
–	Pronotal carinae absent	**98**
72	Female: unknown. Male: radial cell 1.8× as long as wide; club shape begins at F2 and rhinaria at F3; F1 longer than pedicel and F2, F2 longer than F3 (Fig. 13[15]); all flagellomeres straight; propodeal carinae with curved sides	***A.brevitarsis* (Thomson, 1862)**
–	Radial cell > 1.8× as long as wide; without combination of characters as above	**73**
73	Rhinaria and club shape begin at different flagellomeres	**74**
–	Rhinaria and club shape begin at the same flagellomeres	**75**
74	Female: club shape begins at F2 and rhinaria at F1, F2 subequal to F3 (Fig. 15[31]); apex of scutellum without carinae; propodeal carinae form a wide plate with curved sides; radial cell 2.6× as long as wide (Fig. 19[15]). Male: unknown	***A.xanthocera* (Thomson, 1862)**
–	Female: club shape begins at F3 and rhinaria at F4, F2 shorter than F3. Male: rhinaria and club shape begin at F2, F2 subequal to F3 (Fig. 14[5]); apex of scutellum has a thick carina; propodeal carinae separated on top, forming a plate on the bottom with curved sides	***A.hansoni* (Pujade-Villar, 2011)**
75	F1 longer than pedicel	**76**
–	F1 shorter than or subequal to pedicel	**85**
76	Propodeal carinae independent	**77**
–	Propodeal carinae form a plate	**79**
77	Female: rhinaria and club shape begin at F4 (Fig. 13[10]). Male: unknown	***A.australiae* (Ashmead, 1900)**
–	Female: unknown. Male: rhinaria and club shape begin at F1	**78**
78	Male: rhinaria and club shape begin in the last three-quarters of F1 (Fig. 15[33])	***A.xanthopsis* (Ashmead, 1896)**
–	Male: rhinaria and club shape both begin at F1 (Fig. 14[8])	***A.japonicus* (Ashmead, 1904)**
79	Rhinaria and club shape begin at F2 in both sexes; F1–F4 subequal in length (Fig. 13[27]); radial cell 2.3× as long as wide in females (Fig. 17[25]), 2.6× as long in males	***A.evenhuisi* (Ferrer-Suay & Pujade-Villar, 2012)**
–	Rhinaria and club shape begin at F3 or F4 in females, F1 or F2 in males; F1–F4 unequal in length; variety of radial cell sizes	**80**
80	Rhinaria and club shape begin at F4 in females and at F1 in males (when known)	**81**
–	Rhinaria and club shape begin at F3 in females, F1 or F2 in males	**82**
81	Female: radial cell 2.4× as long as wide (Fig. 13[3]). Male: club shape begins at F1 and rhinaria at F3; F1 longer than F2, F2 subequal to F3, F3 shorter than F4; radial cell 2.7× as long as wide (Fig. 17[3]); propodeal carinae well defined, separated by setae on the first half and forming a plate on the last half, straight sides	***A.antananarivoi* (Ferrer-Suay & Pujade-Villar, 2012)**
–	Female: radial cell 2.7× as long as wide (Fig. 14[24]). Male: unknown. Propodeal carinae forming a complete plate with few setae on top and divergent peaks on base	***A.nigricans* (Hellén, 1963)**
82	F1 longer than F2, F2 subequal to F3, F3 shorter than F4 (Fig. 14[23]). Male: F1–F4 subequal; radial cell 2.9× as long as wide in females (Fig. 18[14]) and 2.7× as long as wide in males	***A.nepalica* (Ferrer-Suay & Pujade-Villar, 2013)**
–	With a different combination of characters	**83**
83	Propodeal carinae independent, thin on top and bottom, with curved sides; F1–F3 subequal (Fig. 16[6]); radial cell 2.5× as long as wide (Fig. 19[24])	***A.neartica* (Ferrer-Suay & Pujade-Villar, 2014)**
–	Propodeal carinae form a plate; F1–F3 unequal; radial cell less than 2.5× as long as wide	**84**
84	Female: F2 shorter than F3 (Fig. 15[13]). Male: rhinaria begins at F1; F3 subequal to pedicel, which is slightly curved; propodeal plate with slightly curved sides; radial cell 2.7× as long as wide in females (Fig. 18[29]) and 2.4× as long as wide in males	***A.pusilla* (Kieffer, 1902)**
–	Female: F2 subequal to F3 (Fig. 15[8]). Male: rhinaria begins at F2; F3 unequal to pedicel and no curved flagellomere; propodeal plate with curved sides; radial cell 2.4× as long as wide (Fig. 18[24])	***A.pilipennis* (Hartig, 1840)**
85	Rhinaria and club shape begin at F5 in females and F4 in males. Female: F1 subequal to pedicel and longer than F2, F2 shorter than F3, F3 shorter than F4. Male: F1 shorter than pedicel. Two propodeal carinae, narrow, well defined at upper half, wide and forming a plate on the lower half, with sharp edges	***A.sawoniewiczi* (Kierych, 1988)**
–	Female: rhinaria and club shape begin at different flagellomeres; without combination of characters as above	**86**
86	F1 shorter than pedicel	**87**
–	F1 subequal to pedicel	**88**
87	Rhinaria and club shape begin at F5; F1–F3 subequal in length, F3 shorter than F4 (Fig. 14[7]); radial cell 1.9× as long as wide (Fig. 17[30])	***A.heptatoma* (Hellén, 1963)**
–	Rhinaria and club shape begin at F1; F1 slightly shorter than F2, F2–F4 subequal in length (Fig. 16[8]); radial cell 2.8× as long as wide (Fig. 19[26])	***A.petchabunensis* (Ferrer-Suay & Pujade-Villar, 2014)**
88	Rhinaria and club shape begin at F4; F1 longer than F2, F2 subequal to F3 (Fig. 15[15]); pronotum with two small carinae (sometimes difficult to see), separated by setae in the first third, forming a plate in the last two-thirds; radial cell 2.0× as long as wide (Fig. 18[31])	***A.ramulifera* (Thomson, 1862)**
–	Rhinaria and club shape begin at F3; without combination of characters as above	**89**
89	Antennae shorter than body length; flagellomeres short and round; radial cell 2.0× as long as wide, short and round (Fig. 15[21])	***A.sarae* (Ferrer-Suay, 2012)**
–	Antennae longer than body length; flagellomeres elongated; radial cell more than 2.0× as long as wide	**90**
90	Female: F1 longer than F2, F2 subequal to F3, F3 shorter than F4 (Fig. 13[7]). Male: F1 longer than pedicel and subequal to F2, F2 slightly curved and shorter than F3; propodeum with curved sides; radial cell 2.3× as long as wide (Fig. 17[6])	***A.arcuata* (Kieffer, 1902)**
–	F1 longer than F2, F2–F4 subequal in length (Fig. 13[24]); propodeal plate with slightly curved sides; radial cell 2.6× as long as wide (Fig. 17[22]). Male: unknown	***A.crassicornis* (Thomson, 1862)**
91	Head yellow; F1 longer than F2, F2–F4 subequal (Fig. 15[30]); radial cell 3.0× as long as wide (Fig. 19[14]); propodeum without setae where usually present in other species	***A.victrix* (Westwood, 1833)**
–	Head brown; without combination of characters as above	**92**
92	Pronotal carinae absent	**93**
–	Pronotal carinae present	**95**
93	F1–F3 unequal in length	***A.kovilovica* (Ferrer-Suay & Pujade-Villar, 2013)**
–	F1–F3 subequal in length	**94**
94	Female: rhinaria and club shape begin at F4; F1 longer than pedicel (Fig. 13[5]); radial cell 2.4× as long as wide (Fig. 17[5]). Male: with antennae unknown	***A.aperta* (Hartig, 1841)**
–	Female: unknown. Male: rhinaria and club shape begin at F3; F1 subequal to pedicel (Fig. 15[14]); radial cell 2.5× as long as wide (Fig. 17[30])	***A.quedenfeldti* (Kieffer, 1909)**
95	Female: rhinaria and club shape begin at F3; F1 longer than pedicel, F1 subequal to F2, F2 shorter than or subequal to F3 (Fig. 13[18]). Male: F1–F3 not curved	***A.circumscripta* (Hartig, 1841)**
–	Rhinaria and club shape begin in other flagellomere; without combination of characters as above	**96**
96	Female: rhinaria and club shape begin at F2; F2 shorter than F3 (Fig. 14[11]). Male: F1 curved; radial cell 2.0× as long as wide (Fig. 18[2])	***A.leunisii* (Hartig, 1841)**
–	Female: rhinaria and club shape begin at F3 or F4; F2 subequal to F3 (Fig. 13[22]). Male: F1–F3 curved; radial cell 2.7× as long as wide (Fig. 17[20])	**97**
97	Often found attacking *Brevicorynebrassicae* (L., 1758) through *Diaeretiellarapae* (M’Intosh, 1855)	***A.consobrina* (Zetterstedt, 1838)**
–	Often found attacking *Cryptomizus* sp. through *Aphidiusribis* (Haliday, 1834)	***A.tscheki* (Giraud, 1860)**
98	F1 longer than pedicel	**99**
–	F1 shorter than or subequal to pedicel	**103**
99	Propodeal carinae straight and independent, widening at the base. Female: rhinaria and club shape begin at F2; F1 longer than pedicel and F2, F2–F4 subequal in length (Fig. 14[10]). Male: rhinaria and club shape begin at F1; F1 longer than pedicel and F2, F2–F4 subequal in length; radial cell 2.5× as long as wide (Fig. 18[1])	***A.lachni* (Ashmead, 1885)**
–	Propodeal carinae with different morphology; rhinaria and club shape begin at different flagellomeres; various flagellomere proportions; radial cell < 2.5× as long as wide	**100**
100	Female: rhinaria and club shape begin at F3; F1–F3 subequal in length, F3 shorter than F4 (Fig. 14[1]). Male: rhinaria and club shape begin at F3; F1–F3 subequal in length, F3 shorter than F4; F3 curved; propodeal carinae separated and well-defined; radial cell 2.2× as long as wide (Fig. 17[26])	***A.fracticornis* (Thomson, 1862)**
–	Flagellomere proportions differ in males and females; propodeal carinae form a plate; radial cell shorter or longer than, not equal to 2.2× as long as wide	**101**
101	Female: unknown. Male: rhinaria and club shape begin at F1; F2 longer than F3, F3 subequal to F4 (Fig. 14[19]); propodeal carinae form a plate with slightly-curved sides and few setae on top; radial cell 2.1× as long as wide (Fig. 18[10])	***A.mexicana* (Ferrer-Suay & Pujade-Villar, 2012)**
–	Male: unknown. Female: rhinaria and club shape begin at F3; F2 subequal to F3, F3 shorter than F4; propodeal carinae form a plate with sides that are slightly curved or have two peaks; radial cell shorter or longer than, not equal to 2.1× as long as wide	**102**
102	Propodeal carinae form a plate; radial cell 1.8× as long as wide (Fig. 15[27]). Male: unknown	***A.torresi* (Ferrer-Suay & Pujade-Villar, 2012)**
–	Propodeal carinae thin and straight on top, forming a plate on bottom, with peaks on the sides; radial cell 2.2× as long as wide (Fig. 16[29]). Male: unknown	***A.hendrickxi* (Benoit, 1956)**
103	F1 subequal to pedicel; F1 longer than F2, F2 subequal to F3 (Fig. 1[22])	***A.mullensis* (Cameron, 1883)**
–	F1 shorter than pedicel, F1–F3 subequal in length, F1 sometimes slightly longer	**104**
104	Antennae shorter than body length; forewing with normal marginal setae (the lenght that usually have all other species)	***A.brevis* (Thomson, 1862)**
–	Antennae subequal or longer than body length; forewing with long marginal setae (longer than the lenght that usually have all other species)	***A.darci* (Girault, 1933)**

**Figure 13.**
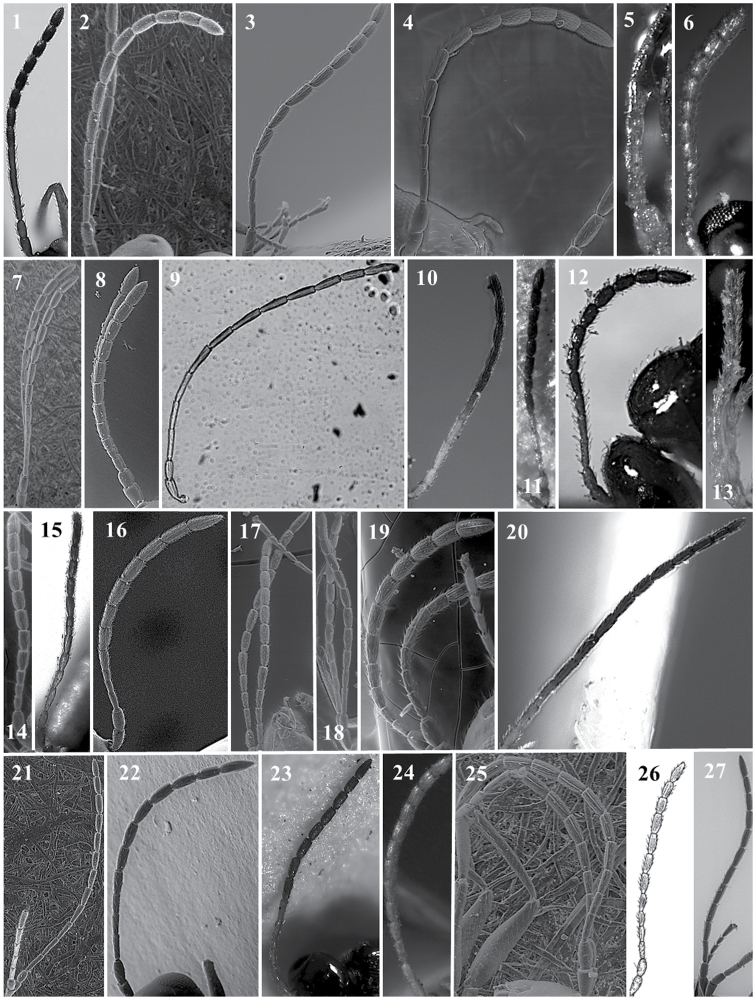
*Alloxysta* antennae. *A.abdera* (**1**); *A.andrewsi* (**2**); *A.antananarivoi* (**3**); *A.antsirananae* (**4**); *A.aperta* (**5**); *A.apteroidea* (**6**); *A.arcuata* (**7**); *A.asiatica* (**8**); *A.aurata* (**9**); *A.australiae* (**10**); *A.basimacula* (**11**); *A.brachycera* (**12**); *A.brachyptera* (**13**); *A.brevis* (**14**); *A.brevitarsis* (**15**); *A.carinata* (**16**); *A.castanea* (**17**); *A.circumscripta* (**18**); *A.citripes* (**19**); *A.commensuratus* (**20**); *A.costaricensis* (**21**); *A.consobrina* (**22**); *A.crassa* (**23**); *A.crassicornis* (**24**); *A.darci* (**25**); *A.desantisi* (**26**); *A.evenhuisi* (**27**).

**Figure 14.**
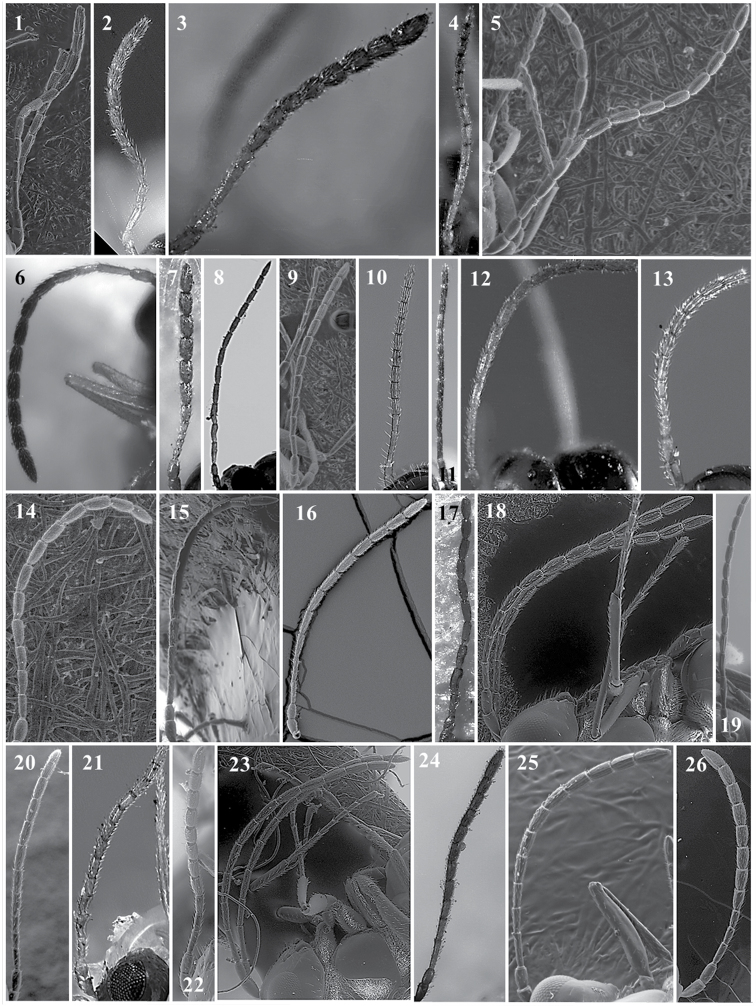
*Alloxysta* antennae. *A.fracticornis* (**1**); *A.fuscipes* (**2**); *A.glebaria* (**3**); *A.halterata* (**4**); *A.hansoni* (**5**); *A.hendrickxi* (**6**); *A.heptatoma* (**7**); *A.japonicus* (**8**); *A.kovilovica* (**9**); *A.lachni* (**10**); *A.leunisii* (**11**); *A.longipennis* (**12**); *A.longiventris* (**13**); *A.luismii* (**14**); *A.macrophadnus* (**15**); *A.mara* (**16**); *A.marshalliana* (**17**); *A.medinae* (**18**); *A.mexicana* (**19**); *A.melanogaster* (**20**); *A.minuscula* (**21**); *A.mullensis* (**22**); *A.nepalica* (**23**); *A.nigricans* (**24**); *A.nigrita* (**25**); *A.nipona* (**26**).

**Figure 15.**
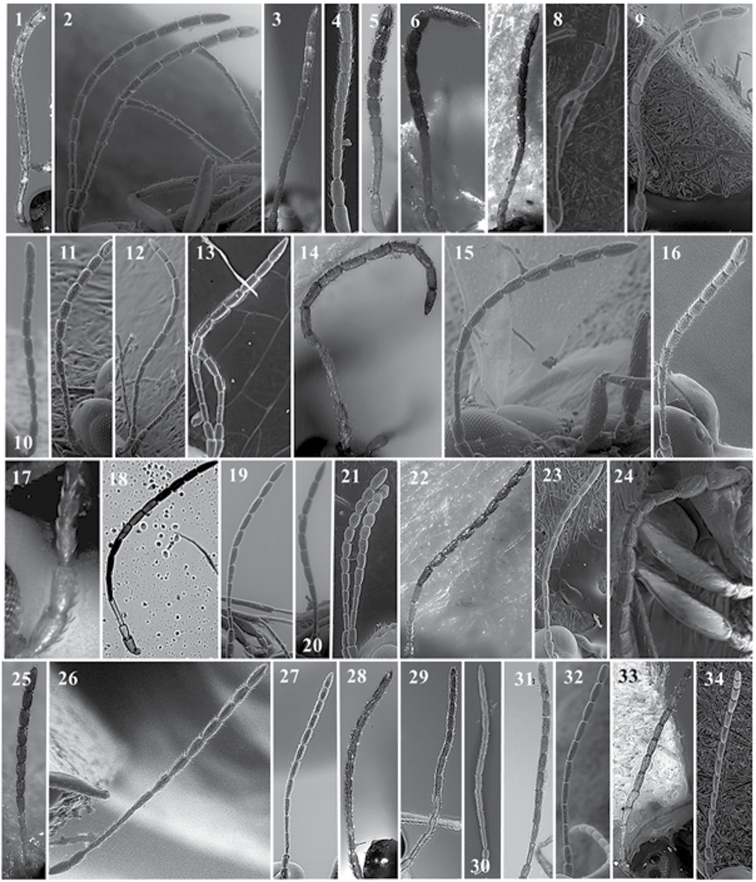
*Alloxysta* antennae. *A.nothofagi* (**1**); *A.obscurata* (**2**); *A.pallidicornis* (**3**); *A.paretasmartinezi* (**4**); *A.patens* (**5**); *A.pedestris* (**6**); *A.piceomaculata* (**7**); *A.pilipennis* (**8**); *A.pilosa* (**9**); *A.pleuralis* (**10**); *A.postica* (**11**); *A.proxima* (**12**); *A.pusilla* (**13**); *A.quedenfeldti* (**14**); *A.ramulifera* (**15**); *A.rubidus* (**16**); *A.rufiventris* (**17**); *A.salicicola* (**18**); *A.sawoniewiczi* (**19**); *A.samurai* (**20**); *A.sarae* (**21**); *A.semiaperta* (**22**); *A.sharkey* (**23**); *A.slovenica* (**24**); *A.soluta* (**25**); *A.thorpei* (**26**); *A.torresi* (**27**); *A.tscheki* (**28**); *A.vandenboschi* (**29**); *A.victrix* (**30**); *A.xanthocera* (**31**); *A.xanthopa* (**32**); *A.xanthopsis* (**33**); *A.centroamericana* (**34**).

**Figure 16.**
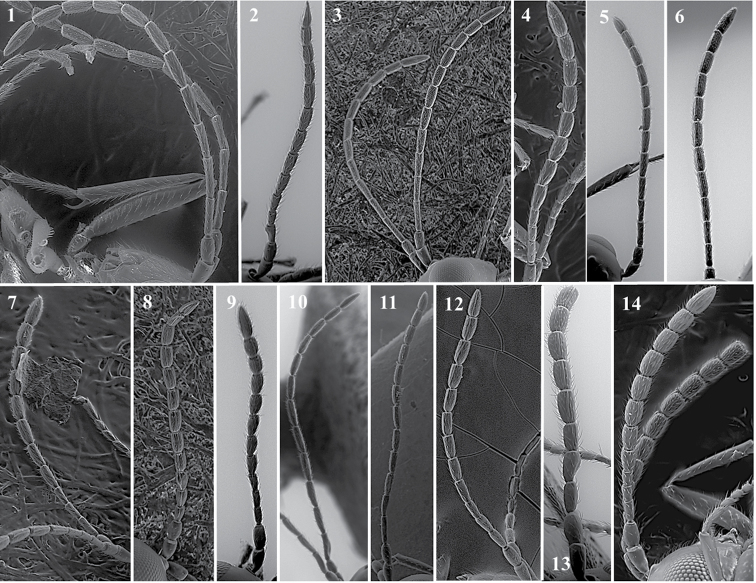
*Alloxysta* antennae. *A.alpina* (**1**); *A.areeluckator* (**2**); *A.buffingtoni* (**3**); *A.franca* (**4**); *A.huberi* (**5**); *A.neartica* (**6**); *A.pili* (**7**); *A.petchabunensis* (**8**); *A.texanae* (**9**); *A.vicenti* (**10**) ; *A.viellae* (**11**); *A.corta* (**12)**; *A.palearctica* (**13**); *A.pascuali* (**14**); *A.nottoni* (**15**).

**Figure 17.**
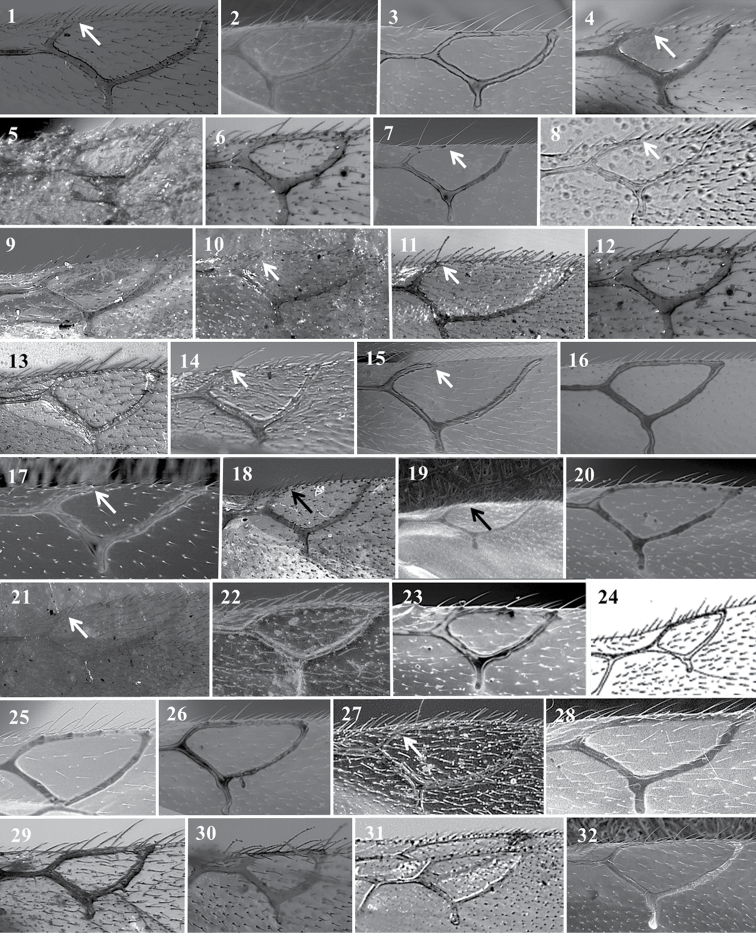
*Alloxysta* radial cell. *A.abdera* (**1**); *A.andrewsi* (**2**); *A.antananarivoi* (**3**); *A.antsirananae* (**4**); *A.aperta* (**5**); *A.arcuata* (**6**); *A.asiatica* (**7**); *A.aurata* (**8**); *A.australiae* (**9**); *A.basimacula* (**10**); *A.brachycera* (**11**); *A.brevis* (**12**); *A.brevitarsis* (**13**); *A.carinata* (**14**); *A.castanea* (**15**); *A.circumscripta* (**16**); *A.citripes* (**17**); *A.commensuratus* (**18**); *A.costaricensis* (**19**); *A.consobrina* (**20**); *A.crassa* (**21**); *A.crassicornis* (**22**); *A.darci* (**23**); *A.desantisi* (**24**); *A.evenhuisi* (**25**); *A.fracticornis* (**26**); *A.fuscipes* (**27**); *A.hansoni* (**28**); *A.hendrickxi* (**29**); *A.heptatoma* (**30**); *A.japonicus* (**31**); *A.kovilovica* (**32**).

**Figure 18.**
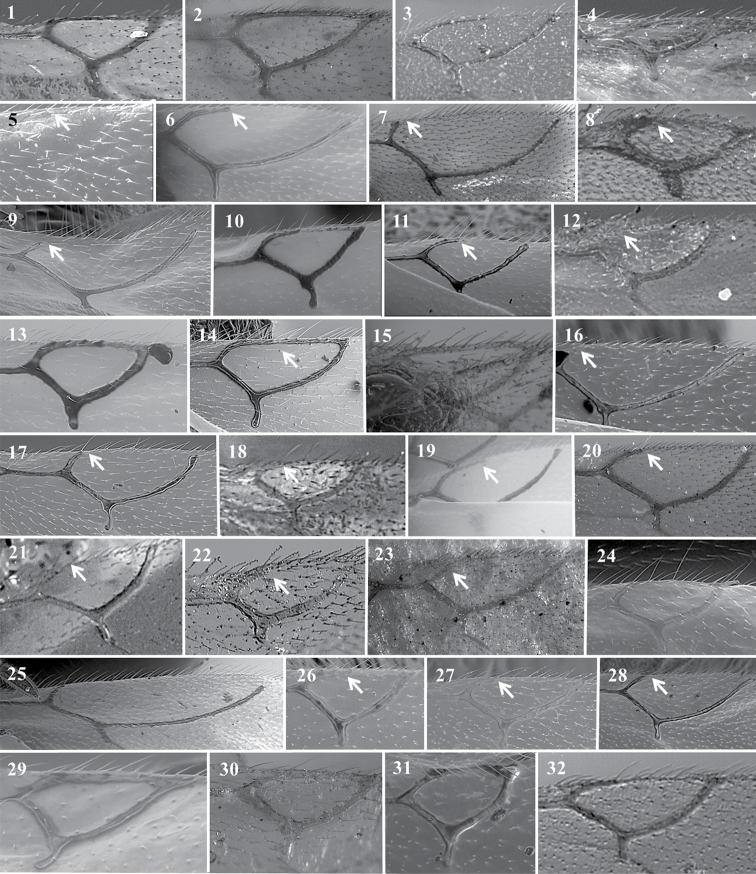
*Alloxysta* radial cell. *A.lachni* (**1**); *A.leunisii* (**2**); *A.longipennis* (**3**); *A.longiventris* (**4**); *A.luismii* (**5**); *A.macrophadna* (**6**); *A.mara* (**7**); *A.marshalliana* (**8**); *A.medinae* (**9**); *A.mexicana* (**10**); *A.melanogaster* (**11**); *A.minuscula* (**12**); *A.mullensis* (**13**); *A.nepalica* (**14**); *A.nigricans* (**15**); *A.nigrita* (**16**); *A.nipona* (**17**); *A.nothofagi* (**18**); *A.obscurata* (**19**); *A.pallidicornis* (**20**); *A.paretasmartinezi* (**21**); *A.patens* (**22**); *A.piceomaculata* (**23**); *A.pilipennis* (**24**); *A.pilosa* (**25**); *A.pleuralis* (**26**); *A.postica* (**27**); *A.proxima* (**28**); *A.pusilla* (**29**); *A.quedenfeldti* (**30**); *A.ramulifera* (**31**); *A.rubidus* (**32**).

**Figure 19.**
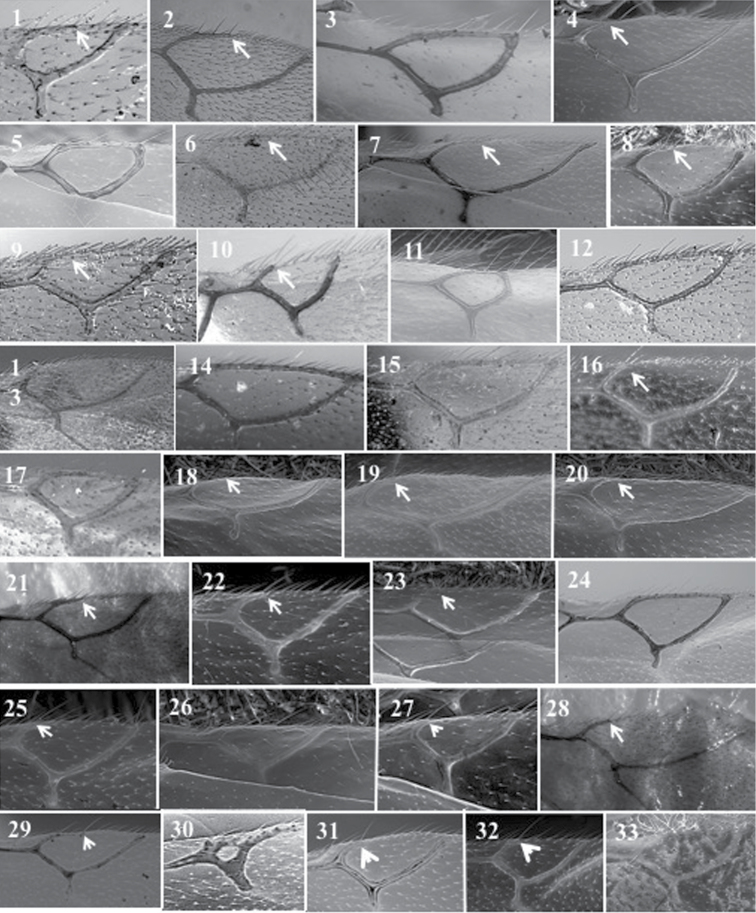
*Alloxysta* radial cell. *A.rufiventris* (**1**); *A.salicicola* (**2**); *A.sawoniewiczi* (**3**); *A.samurai* (**4**); *A.sarae* (**5**); *A.semiaperta* (**6**); *A.sharkey* (**7**); *A.slovenica* (**8**); *A.soluta* (**9**); *A.thorpei* (**10**); *A.torresi* (**11**); *A.tscheki* (**12**); *A.vandenboschi* (**13**); *A.victrix* (**14**); *A.xanthocera* (**15**); *A.xanthopa* (**16**); *A.xanthopsis* (**17**); *A.centroamericana* (**18**); *A.alpina* (**19**); *A.areeluckator* (**20**); *A.buffingtoni* (**21**); *A.franca* (**22**); *A.huberi* (**23**); *A.neartica* (**24**); *A.pili* (**25**); *A.petchabunensis* (**26**); *A.texanae* (**27**); *A.vicenti* (**28**); *A.viellae* (**29**); *A.curta* (**30**); *A.palearctica* (**31**); *A.pascuali* (**32**); *A.nottoni* (**33**).

#### Apocharips

Taxon classificationAnimaliaHymenopteraFigitidae

Fergusson, 1986

Apocharips Fergusson, 1986: 16. Type: Allotriaxanthocephala Thomson, 1862.

##### General features.

**Figure 20.**
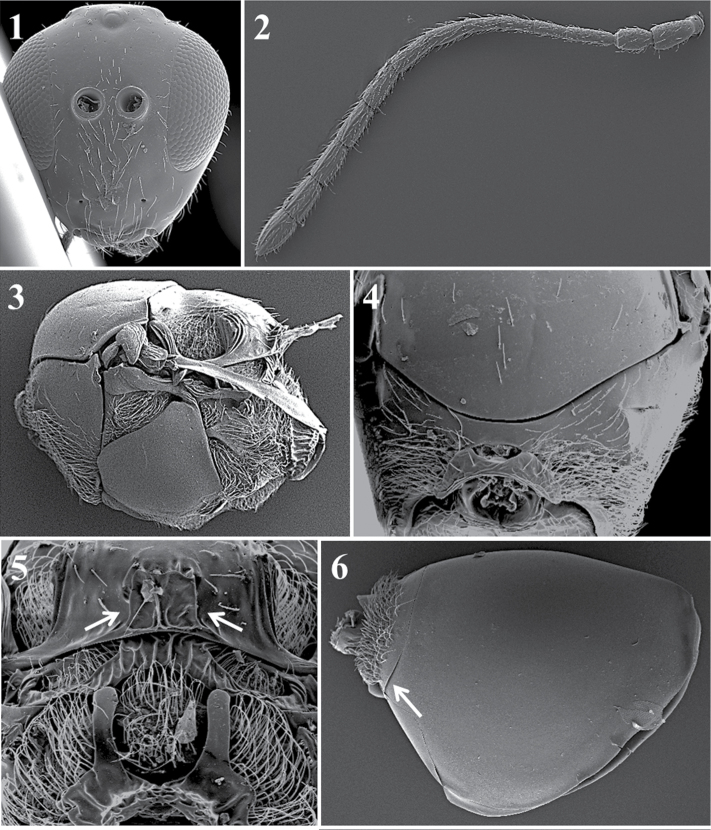
*Apocharips* general features. Head (**1**); antenna (**2**); mesosoma (**3**); pronotum (**4**); propodeum (**5**); metasoma (**6**).

*Head*. Triangular, higher than it is wide, smooth and shiny. Setae present below and between toruli with few setae above toruli. Scattered setae on vertex, many setae on frons. Transfacial line 0.9–0.8× the height of the compound eye. Malar space 0.3–0.4 × the height of the compound eye (Fig. 20[1]).

*Antenna*. Female: 13-segmented, filiform. All antennomers covered with sparse setae (Fig. 20[2]). Male: 14-segmented, filiform. All antennomers covered with sparse setae.

*Mesosoma*. Pronotum with setae; two thick, curved, long carinae (Fig. 20[4]). Mesoscutum smooth and shiny, round in dorsal view with sparse setae. Scutellum smooth and shiny with scattered setae, an M-shaped carina on the apex of scutellum. Propodeum with abundant setae; two propodeal carinae separated by setae in first third, forming a plate in last two-thirds, with strongly-curved sides (Fig. 20[5]).

*Forewing*. Longer than the body, 1.1–1.5× as long as the mesosoma and metasoma combined, with dense pubescence and marginal setae. Open radial cell in variable sizes. Shape of R1 and Rs veins vary.

*Metasoma*. Has a small basal metasomal tergum, terminating just posterior to the ring of setae at the base of the metasoma (Fig. 20[6]).

##### Distribution.

Holarctic, Neotropical and African regions (Ferrer-Suay et al. 2013).

##### Biology.

Endoparasitoids of Encyrtidae (Hymenoptera: Chalcidoidea) that are endoparasitoids of psyllids (Hemiptera: Psyllidae) (Fergusson 1986; Menke and Evenhuis 1991). Until now it has been cited in: *Euphylluraolivine* and *Euphylluraaethiopica* by Silvestri (1915: 274).

### Key to species

**Table:** 

1	Radial cell is short, 1.2× as long as wide, with parallel R1 and Rs (Fig. 22[5])	***A.trapezoidea* (Hartig, 1841)**
–	Radial cell is longer, 2.0–2.8× as long as wide; R1 and Rs non-parallel	**2**
2	Lower face with small radial carinae around clypeus	***A.hansoni* Menke, 1993**
–	Face smooth, without carinae	**3**
3	Last two flagellomeres well-differentiated, not broadly joined	**4**
–	Last two flagellomeres broadly joined	**5**
4	Many long setae under toruli. Female: rhinaria and club shape (antenna widening) begin at F5; F1 longer than F2 which is subequal to F3 (Fig. 21[1]); propodeal carinae are thick and separated; apex of scutellum has carinae (Fig. 22[1]). Male: unknown	***A.angelicae* (Pujade-Villar & Evenhuis, 2002)**
–	Short, scattered setae under toruli. Female: rhinaria and club shape begin at F2; F1 = F2 < F3 (Fig. 21[2]); propodeal carinae form a plate with strongly curved margins; apex of scutellum does not have carinae (Fig. 22[2]). Male: unknown	***A.colombiana* (Ferrer-Suay & Pujade-Villar, 2013)**
5	Male: rhinaria and club shape begin at F4; F1 = F2 < F3 (Fig. 21[6]); Rs is curved, reaching wing margin (Fig. 22[6]). Female: unknown	***A.tropicale* (Ferrer-Suay & Paretas-Martínez, 2013)**
–	Male: rhinaria and club shape begin at F5; F1 > F2 = F3 (Fig. 21[4]); Rs is straight and does not reach wing margin (Fig. 22[4]). Female: unknown	***A.tamanii* (Paretas-Martínez & Pujade-Villar, 2013)**

**Figure 21.**
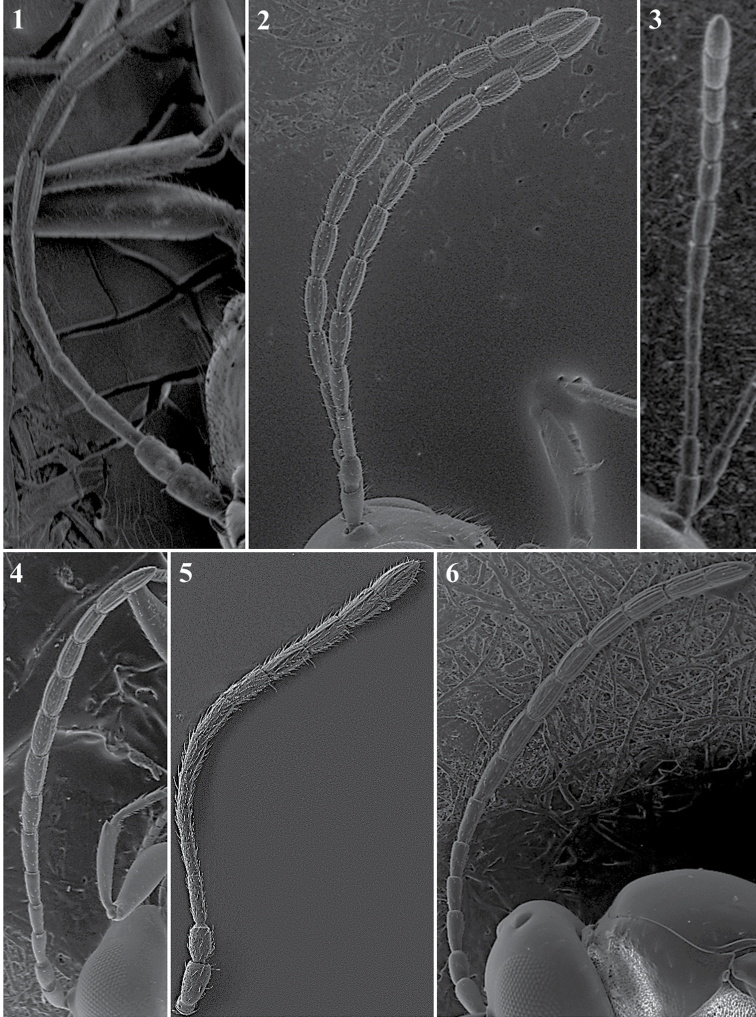
*Apocharips* antennae. *A.angelicae* (**1**); *A.colombiana* (**2**); *A.hansoni* (**3**); *A.tamani* (**4**); *A.trapezoidea* (**5**); *A.tropicale* (**6**).

**Figure 22.**
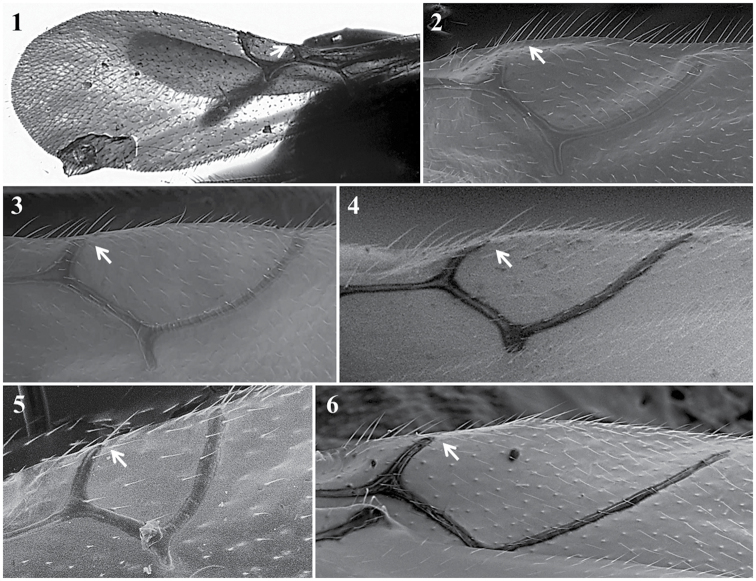
*Apocharips* radial cell. *A.angelicae* (**1**); *A.colombiana* (**2**); *A.hansoni* (**3**); *A.tamanii* (**4**); *A.trapezoidea* (**5**); *A.tropicale* (**6**).

#### Dilapothor

Taxon classificationAnimaliaHymenopteraFigitidae

Paretas-Martínez & Pujade-Villar, 2006

Dilapothor Paretas-Martinez & Pujade-Villar, 2006: 224. Type: Dilapothorcarverae Paretas-Martínez & Pujade-Villar, 2006.

##### General features.

**Figure 23.**
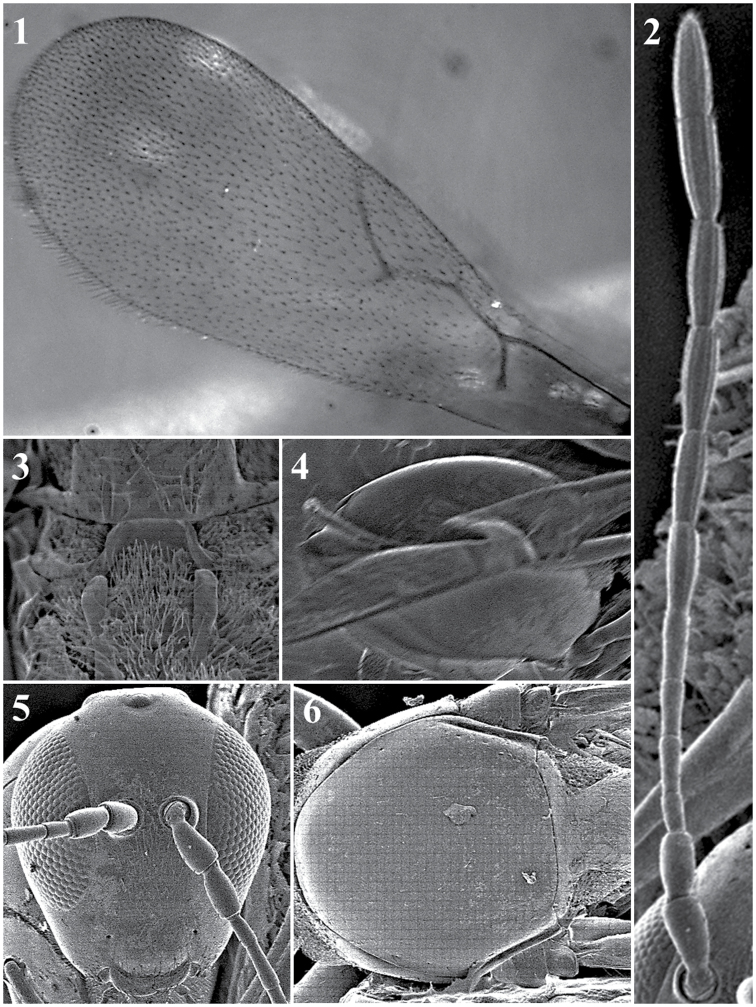
*Dilapothorcarverae* Paretas-Martínez and Pujade-Villar, 2006. Fore wing (**1**); antenna (**2**); propodeum (**3**); metasoma (**4**); head (**5**); mesoscutum (**6**).

*Head*. Elongated in anterior view, eyes located at the higher part of the head, malar space is more than double the distance from the external margin of the lateral ocellus to the dorsal margin of the compound eye, measured in the anterior view of the head. With some setae below the toruli; sparse setae on frons (Fig. 23[5]).

*Antenna*. Female: 12-segmented, clavate. Flagellomeres separate except the last two, which are broadly joined, all antennomers are covered with sparse setae. Each ﬂagellomere expands towards its distal end (Fig. 23[2]).

*Mesosoma*. Pronotal carinae small, only slightly indicated. Mesoscutum is smooth, shiny, and is almost completely without setae (Fig. 23[6]). No sutures on the mesopleuron. Scutellum has three carinae on each side of the scutellar apex that are symmetrical, with a distance between them equivalent to the distance between the propodeal carinae. Propodeum has two strong, broad carinae (Fig. 23[3]).

*Forewing*. Large, longer than body and covered with dense pubescence; marginal setae are present, but not very long. Large radial cell is completely open; 2r as long as Sc + R1; Rs are long and curved, giving an elongated, large appearance to radial cell; R1 is very short and does not reach the costal margin; 2rm is very short, almost absent; Cu1a, M + Cu1a, Rs + M and M veins absent (Fig. 23[1]).

*Metasoma*. Proximal part of metasoma has a complete ring of setae. Metasoma not segmented, only one big tergite visible (Fig. 23[4]).

##### Comments.

Until now there is only one species known of this genus, *Dilapothorcarverae* Paretas-Martínez & Pujade-Villar, 2006.

##### Distribution.

Only known from Australia (Paretas-Martínez and Pujade-Villar 2006).

##### Hosts.

Unknown (Paretas-Martínez and Pujade-Villar 2006).

#### Dilyta

Taxon classificationAnimaliaHymenopteraFigitidae

Förster, 1869

Dilyta Förster, 1869: 340. Type: Dilytasubclavata Förster, 1869: 340.
Dylita Förster, 1869: 338. An incorrect original spelling (rejected by Menke and Evenhuis 1991:152, first revisers), unavailable.
Charips Haliday in Marshall, 1870: 181. Type: Charipsmicrocera Haliday in Marsall, 1870. Synonymized by Hellén (1963: 4).
Allotria (Glyptoxysta Thomson, 1877: 881).
Glyptoxysta Thomson, 1877 in Ashmead (1903: 142). Type: Glyptoxystaheterocera Thomson, 1877. Synonymized by Hellén (1963: 4).

##### General features.

**Figure 24.**
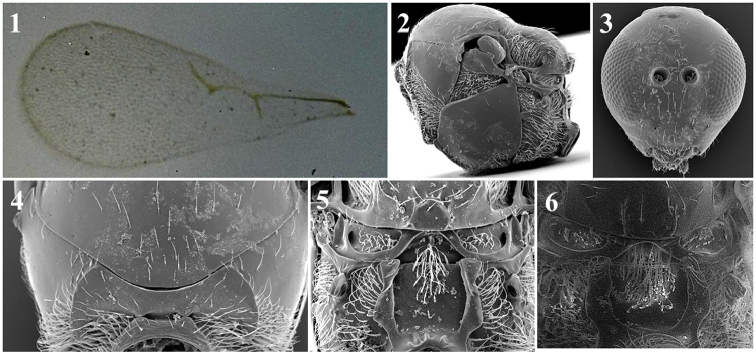
*Dilyta* general features. Forewing (**1**); mesosoma (**2**); head (**3**); pronotum (**4**); propodeum holarctic species (**5**); propodeum african species (**6**).

*Head*. Rounded in anterior view, eyes located at middle line of head, malar space subequal to the distance from external margin of the lateral ocellus to the dorsal margin of the compound eye, measured in anterior view of the head. Surface completely smooth, without any strigose, malar impression, epistomal sulcus or clypeo-pleurostomal lines. Clypeus almost straight, slightly projecting over mandibles, without marginal inflection. Setae sparse, concentrated principally below the toruli (Fig. 24[3]).

*Antennae*. Size of pedicel and flagellomeres vary among species. Female: 13-segmented, slightly clavate; two last segments (F10–F11) broadly jointed. Male: 14-segmented, slightly clavate or filiform; two last segments (F11–F12) broadly jointed.

*Mesosoma*. Pronotum have setae only in the anterior part; pronotal carinae is long, clearly indicated, and extends from scutum to the anterior part of pronotum (Fig. 24[4]). Mesoscutum smooth, shiny, and almost without setae. Mesopleuron smooth, without any longitudinal ridge in lower part (Fig. 24[2]). Scutellum smooth, with scarce setae at posterior and lateral parts. Propodeum with two strong, broad carinae. Apex of scutellum: *Holarctic* spp. ∩-shaped carina (Fig. 24[5]). *Afrotropical* spp. with one carina on each side, both symmetrical and parallel, with a distance between them equivalent to the distance between the propodeal carinae (Fig. 24[6]).

*Forewing*. Large, longer than body, covered with dense pubescence; marginal, long setae present; brown veins; radial cell small and completely open along anterior margin; R1 very short and barely reaches costal margin (Fig. 24[1]).

##### Distribution.

Holarctic, Afrotropical and Oriental regions (Paretas-Martínez et al. 2011).

##### Hosts.

Endoparasitoids of Encyrtidae (Hymenoptera: Chalcidoidea) that are endoparasitoids of psyllids (Hemiptera: Psyllidae) (Fergusson 1986; Menke and Evenhuis 1991). Until now it has been cited in: *Cacopsyllaalba*, *Cacopsyllapyricola*, *Psyllapyri*, *Psyllopsisfraxini* by Menke and Evenhuis (1991: 152); *Triozaerytreae* by Paretas-Martínez et al. (2009: 211) and Psyllidae on *Firmianasimplex* by Paretas-Martínez et al. (2011: 34).

### Key to species

**Table:** 

1	Apex of scutellum has one carina on each side, both symmetrical, parallel and higher than axillar strip, distance between them equivalent to distance between the propodeal carinae (Fig. 24[6])	**2**
–	Apex of scutellum a ∩-shaped, projected plate (Fig. 24[5])	**6**
2	Metasoma with a punctuated area on distal part	**3**
–	Metasoma does not have punctures	**4**
3	Female: F1 subequal or slightly longer than pedicel, F2 and F3 much shorter than F1, F4 longer than F3 but shorter than F1; F5–F11 wider than previous segments, antenna slightly clavate from F5; sensilla beginning at F4–F5 (Fig. 25[1]). Male: F1 slightly longer than pedicel, F2 much shorter than F1, F3 shorter than F1 but longer than F2, F4 longer than F3 but shorter than F1, F5 wider than and as long as F1; overall, antenna slightly wider from F5 to F12	***D.africana* (Benoit, 1956)**
–	Female: F1 subequal to pedicel, F2 much shorter than F1, F3 wider than and subequal in length to F1; F3–F11 wider than previous segments; antenna slightly clavate from F3; sensilla beginning on F3 (Fig. 25[4]). Male: unknown	***D.ghanana* (Paretas-Martínez, Pujade-Villar & Melika, 2009)**
4	Female: F1 subequal to pedicel; F2 to F4 each shorter than F1; F5 subequal to F1 in length but wider; F5–F11 wider than previous segments, antenna slightly clavate from F5; sensilla beginning on F5–F6 (Fig. 25[3]). Male: unknown	***D.australafricana* (Paretas-Martínez & Pujade-Villar, 2009)**
–	F1 shorter than pedicel; different combination of flagellomeres	**5**
5	Female: F1 shorter than pedicel, F2 and F3 shorter than F1 but not subquadrate, longer than they are wide, F4 longer than F1; F4–F11 wider than previous segments, antenna slightly clavate from F4; sensilla beginning on F4–F5 (Fig. 25[6]). Male: F1 subequal to pedicel; F2 shorter than F1, F3 shorter than F1 but slightly longer than F2, F4 wider than and as long as F1; antenna slightly wider from F4 to F12	***D.kenyana* (Paretas-Martínez & Pujade-Villar, 2009)**
–	Female: F1–F5 very short, each shorter than pedicel; F1 and F5 subequal in length, F2–F4 subquadrate, as wide as they are long, and shorter than F1; F1–F6 wider than previous segments, antenna slightly clavate from F6; sensilla begin at F6 (Fig. 25[12]). Male: F1 straight; F1–F3 each similar to pedicel, subquadrate, and < 1.5 as long as wide; sensilla abundant on F1	***D.somaliana* (Paretas-Martínez, Pujade-Villar & Evenhuis, 2009)**
6	Metasoma with distinct, visible punctuation on distal half	**7**
–	Metasoma does not have punctures, or at most has very few, scattered punctures on distal half that are not clearly visible	**11**
7	Female: unknown. Male: F1 very long, wide and arched, F1 much longer than pedicel (almost double), F1 longer than F2 and F3 combined, F2 slightly shorter than or subequal to F3, F4 longer than F2 and F3, F4–F12 wider than previous segments; antenna slightly clavate from F4, sensilla begin at F4 (Fig. 25[11])	***D.sinica* (Ferrer-Suay & Paretas-Martínez, 2011)**
–	Male, when known, has different features than given above	**8**
8	Female: F1 very long, thin, almost twice as long as pedicel; F1 longer than F2–F5, F1 nearly as long as F2, F3 and F4 combined (Fig. 25[5]). Male: unknown	***D.japonica* (Paretas-Martínez & Ferrer-Suay, 2011)**
–	F1 similar in length to pedicel, F1 shorter than or subequal to F2 and F3 combined	**9**
9	Female: unknown. Male: rhinaria and club shape begin at F1; F1 slightly curved; F1–F3 subequal (Fig. 25[9])	***D.paretasmartinezi* (Pujade-Villar & Ferrer-Suay, 2012)**
–	Rhinaria and club shape begin in different flagellomeres; F1–F3 unequal	10
10	Female: F1 slightly shorter than or subequal than pedicel, F2 subequal to F3, F4 slightly shorter than F1 but longer than F2 and F3, F1 subequal to F5, F6 longer than F5 (Fig. 25[13]). Male: F1 slightly longer than pedicel; F2 and F3 each shorter than F1; F1 subequal to F4; F4–F12 wider than previous flagellomeres, antenna slightly clavate from F4; sensilla begin at F4	***D.subclavata* (Förster, 1869)**
–	Female: F1 subequal to pedicel or slightly longer, F2 shorter than F3, F3 shorter than F4, F4 shorter than F5, F1 subequal to F5 (Fig. 25[7]). Male: F1 subequal to pedicel, F2 shorter than F1 or F3, F3 subequal to F1; F3–F12 wider than previous flagellomeres; antenna slightly clavate from F3; sensilla begin at F3	***D.longinqua* (Paretas-Martínez & Pujade-Villar, 2011)**
11	Female: F1 narrow, slightly longer or subequal to pedicel; F1 longer than F2, F3 and F4; F2 slightly shorter than or subequal to F3; F4 longer than F2 and F3 (Fig. 15[8]). Male: unknown	***D.orientalis* (Ferrer-Suay & Paretas-Martínez, 2011)**
–	F1 markedly or only slightly shorter than pedicel; flagellomere differently proportioned than as above	**12**
12	Female: F1 almost double the length of F2, F3 and F4; F2 and F3 subequal to F4 but sometimes F2 seems slightly shorter than F3 or F4; F5 longer than F4; F5 longer than F4 but shorter than F1; F7–F11 wider than previous flagellomeres; antenna slightly clavate from F6 (Fig. 25[10]). Male: F1 as long as pedicel; F2 slightly shorter than F3; F4 as long as F1 but thinner; sensilla begin at F4	***D.rathmanae* (Menke & Evenhuis, 1991)**
–	Female: F1 longer than F2, F2 slightly longer than F3 and F4, F3 subequal to F4, F5 longer than F4 but as long as F1; F6–F10 wider than previous flagellomeres; antenna slightly clavate from F5 (female antenna has only 10 flagellomeres) (Fig. 25[2]). Male: F1 longer than pedicel, F2 subequal to F3, F4 wider and as long as F1; sensilla begin at F6	***D.aleevae* (Pujade-Villar & Paretas-Martínez, 2011)**

**Figure 25.**
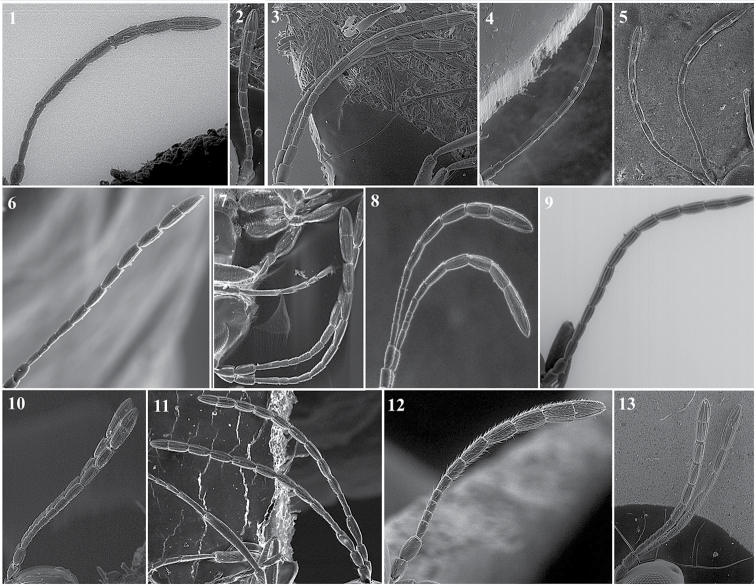
*Dilyta* antennae. *D.africana* (**1**); *D.alevae* (**2**); *D.australafricana* (**3**); *D.ghanana* (**4**); *D.japonica* (**5**); *D.kenyana* (**6**); *D.longinqua* (**7**); *D.orientalis* (**8**); *D.paretasmartinezi* (**9**); *D.rathmanae* (**10**); *D.sinica* (**11**); *D.somaliana* (**12**); *D.subclavata* (**13**).

#### Lobopterocharips

Taxon classificationAnimaliaHymenopteraFigitidae

Paretas-Martínez & Pujade-Villar, 2007

Lobopterocharips Paretas-Martínez et al., 2007: 475. Type: Lobopterocharipsarreplegata Paretas-Martínez & Pujade-Villar, 2007.

##### General features.

**Figure 26.**
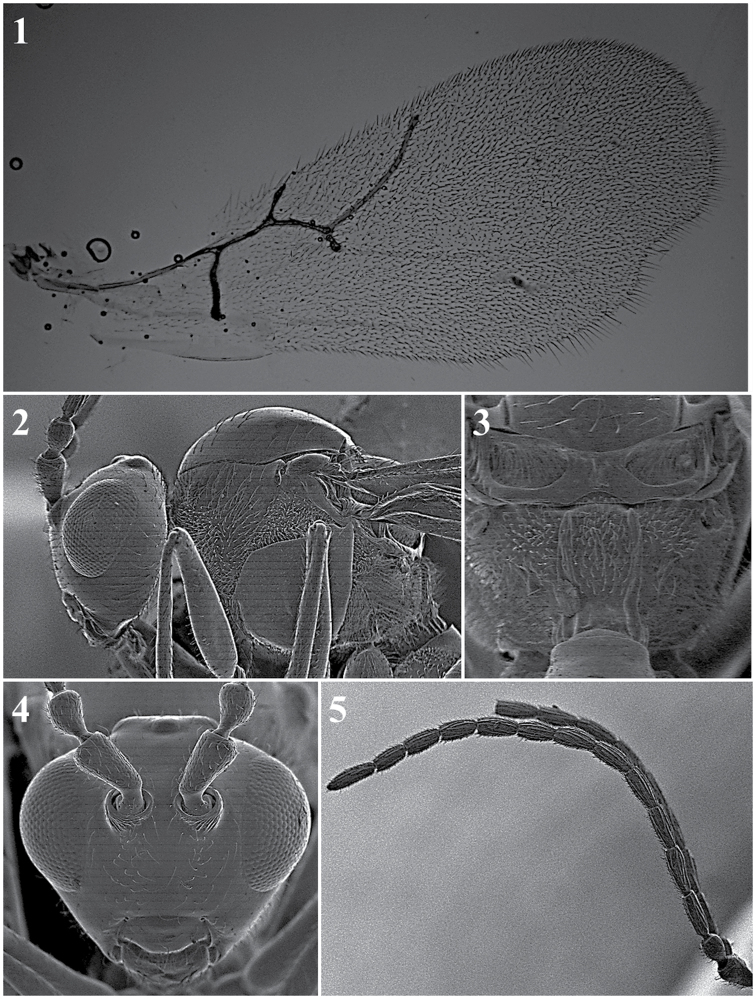
*Lobopterocharipsarreplegata* Paretas-Martinez and Pujade-Villar, 2007. Fore wing (**1**); mesosoma, lateral view (**2**); propodeum (**3**); head, anterior view (**4**); antenna (**5**).

*Head.* Transversely ovate, slightly wider than high in anterior view. Smooth surface, without sculpturing or ridges. Clypeus broadly projected over mandibles, marginal inflection well defined. Epistomal sulcus and clypeo-pleurostomal lines well defined. Toruli wrinkled in inferior area. Malar impression absent (Fig. 26[4]).

*Antennae*. Male: 14-segmented, filiform. Antennomers completely separate, covered with sparse setae. Abundant rhinaria in all flagellomers (Fig. 26[5]).

*Mesosoma*. Pronotal carinae very small, only slightly indicated. Mesopleuron smooth, without wrinkles or furrows (Fig. 26[2]). Mesoscutum smooth, shiny, without notauli or other impressions, and almost with no setae. Parascutal sulcus ending anteriorly next to tegula. Scutellum evenly rounded without distinct sculpture, smooth, no foveae or sculpture on apex. Posterodorsal extensions of axillar strips present. Metascutellum not constricted, with one longitudinal medial carina. Propodeum with two narrow longitudinal carinae (Fig. 26[3]).

*Forewing*. With dense pubescence; marginal setae present. Radial cell completely open, large; 2r as long as Sc+R1; Rs long, curved, ending just before wing margin; R1 reaching wing margin; 2rm well defined; Cu1a, M+Cu1a, Rs+M, M veins present, slightly visible; Rs+M vein pointing to middle part of basal vein. Undulation present in apical part of posterior margin (Fig. 26[1]).

*Metasoma*. Proximal area with incomplete ring of setae, not present in dorsal area. T2-T3 with subequal dorsomedial lengths, covering most of metasoma.

##### Comments.

Until now there is only one species known of this genus, *Lobopterocharipsarreplegata* Paretas-Martinez et al. 2007b.

##### Distribution.

Only known from Nepal (Paretas-Martínez et al. 2007: 475).

##### Hosts.

Unknown (Paretas-Martínez et al. 2007: 475).

#### Lytoxysta

Taxon classificationAnimaliaHymenopteraFigitidae

Kieffer, 1909

Lytoxysta Kieffer, 1909: 479. Type: Lytoxystabrevipalpis Kieffer, 1909.

##### General features.

**Figure 27.**
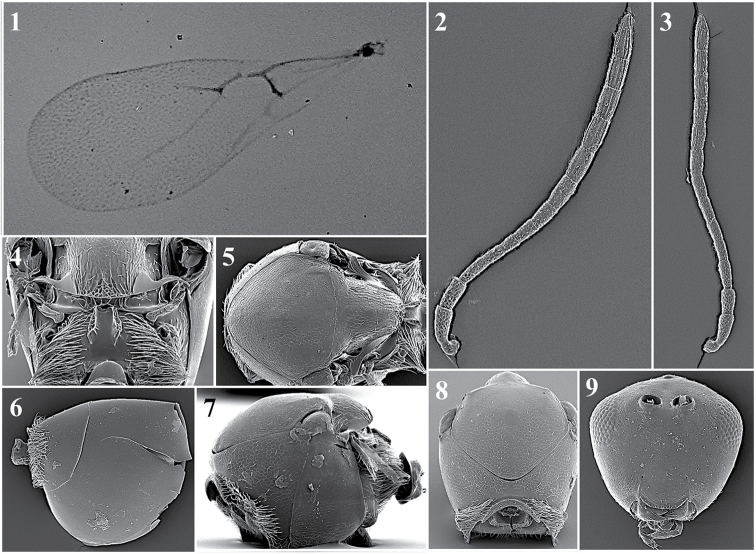
*Lytoxystabrevipalpis* Kieffer, 1909. Fore wing (**1**); antenna female (**2**); antenna male (**3**); propodeum (**4**); mesoscutum (**5**); metasoma (**6**); mesosoa, lateral view (**7**); pronotum (**8**); head, anterior view (**9**).

*Head*. Triangular, higher than it is wide, covered by fine reticulated sculpture. Covered by very few scattered setae (Fig. 27[9]).

*Antenna*. Female: 13-segmented, filiform (Fig. 27[2]). Male: 13-segmented, filiform (Fig. 27[3]).

*Mesosoma*. Entirely covered by fine reticulated sculpture (Fig. 27[7]). Pronotum has no carinae present (Fig. 27[8]). Apex of scutellum has irregular carinae (Fig. 27[5]). Propodeum covered by abundant long setae; two thin and short propodeal carinae on top (Fig. 27[4]).

*Forewing*. Longer than body with dense pubescence and marginal setae. Radial cell is open. R1 and Rs are short and do not reach the costal margin (Fig. 27[1]).

*Metasoma*. Anterior part with an incomplete ring of setae, glabrous at centre and wider laterally. Metasoma smooth and shiny, T3 and T4 clearly distinguished.

##### Comments.

Until now there is only one species known of this genus, *Lytoxystabrevipalpis* Kieffer, 1909.

##### Distribution.

Canada (British Columbia and Manitoba) and USA (California) (Andrews 1978: 24); USA (Massachusetts) (Kieffer 1909: 480).

##### Hosts.

Cited in *Chaitophorussalicicorticis*, *Aphis* sp. and *Dactynotus* sp. throughout *Aphidius* sp. and *Lysiphlebus* sp. (Andrews 1978: 24).

#### Phaenoglyphis

Taxon classificationAnimaliaHymenopteraFigitidae

Förster, 1896

Phaenoglyphis Förster, 1869: 338. Type: Phaenoglyphisxanthochroa Förster, 1869.
Hemicrisis Förster, 1869: 338. Type: Hemicrisisruficornis Förster, 1869. Synonymized by Evenhuis (1973: 218). See the history of placement in Pujade-Villar and Paretas-Martínez (2006).
Glyptoxysta Thomson, 1877: 812. Type: Auloxystanigripes Thomson, 1877, by subsequent designation (Ashmead 1903: 142) (vide Rohwer and Fagan 1919: 237). Synonymized by Hellén (1963: 5).
Bothrioxysta Kieffer, 1902: 11. Type: Auloxystanigripes Thomson, 1877, by subsequent designation (Rohwer and Fagan 1917: 362). Synonymized by Hellén (1963: 5).
Charipsella Brèthes, 1913: 159. Type: Charipsellalaevigata Bréthes, 1913. Synonymized by Quinlan and Evenhuis (1980: 428).

##### General features.

**Figure 28.**
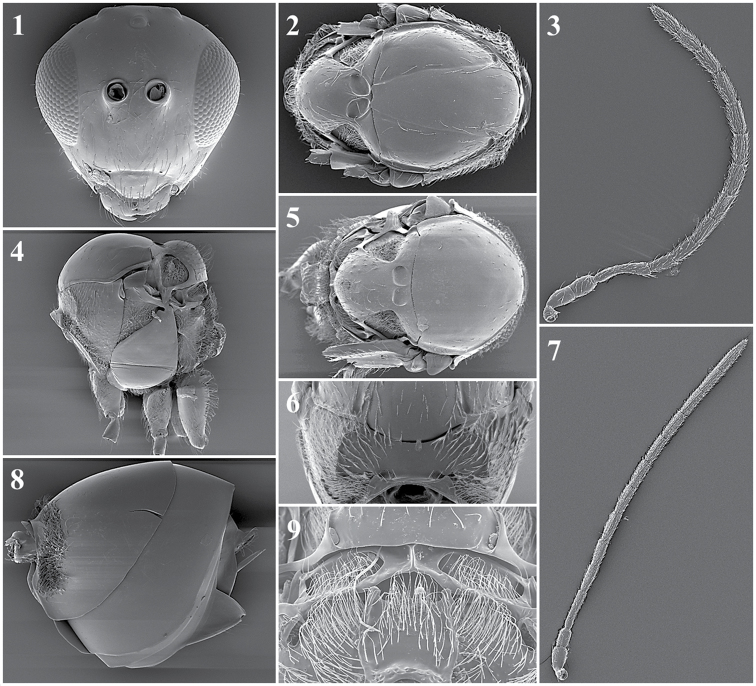
*Phaenoglyphis* general features. Head (**1**); mesoscutum with notauli (**2**); male antenna (**3**); mesosoma (**4**); mesoscutum without notauli (**5**); pronotum (**6**); female antenna (**7**); metasoma (**8**); propodeum (**9**).

*Head*. Transversally ovate, smooth and shiny, slightly wider than it is high from the anterior view. Setae below and between toruli, without setae above toruli. Setae few and scattered on vertex, many setae on face. Transfacial line 1.1–1.2× height of compound eye. Malar space 0.3–0.4× height of compound eye (Fig. 28[1]).

*Antenna*. Female: 13-segmented, filiform. All antennomers have sparse setae (Fig. 28[7]). Male: 14-segmented, filiform. All antennomers have sparse setae (Fig. 28[3]).

*Mesosoma*. Pronotum entirely covered by long setae; two thick and long carinae are clearly visible (Fig. 28[6]). Mesoscutum smooth, shiny and round in dorsal view with scattered setae (Fig. 27[2, 5]). Scutellum smooth and shiny with scattered setae that are more abundant on apex. Propodeum covered with setae; two thin carinae are well-separated (Fig. 28[9]).

*Forewing*. Longer than body, 1.3–1.6× as long as mesosoma and metasoma combined. Covered with dense pubescence; marginal setae present. Radial cell usually closed, very few species with partially or completely open radial cell.

*Metasoma*. Anterior part has an incomplete ring of setae, is glabrous at centre and wider laterally. Metasoma smooth and shiny, T3 and T4 clearly distinguished (Fig. 28[9]).

##### Distribution.

Cosmopolitan (Ferrer-Suay et al. 2012).

##### Hosts.

Endoparasitoids of Aphidiinae (Hymenoptera: Braconidae) and Aphelininae (Hymenoptera: Braconidae) that are endoparasitoids of aphids (Hemiptera: Aphididae) (Fergusson 1986; Menke and Evenhuis 1991).

### Key to species

**Table:** 

1	Notauli present, at least on the posterior half of mesoscutum and/or scutum sculpture (Fig. 28[2])	**2**
–	Notauli completely absent and scutum smooth and shining (Fig. 28[5])	**23**
2	Mesoscutum covered by imbricated sculpture (except mesopleura)	**3**
–	Mesoscutum smooth, without imbricated sculpture	**6**
3	Mesoscutum mostly smooth, with a few wrinkles on the distal side of the notauli	**4**
–	With distinctive imbricate sculpturing on all surfaces	**5**
4	Notaulus very faint; radial cell 2.4× as long as wide	***P.izhizawai* (Watanabe, 1950)**
–	Distinct notaulus; radial cell 2.7× as long as wide	***P.ruficornis* (Förster, 1869)**
5	Rhinaria and club shape begin at F1 (Fig. 29[23]); thick pronotal carinae do not reach mesoscutum; mesoscutum entirely covered by many setae; notauli present, only insinuated on the anterior part and well-marked on the back; scutellar foveae open on the bottom (Fig. 31[23]); central part of metascutellum smooth, with only a central carina. Male: unknown	***P.pubicollis* (Thomson, 1877)**
–	Rhinaria and club shape begin at F4 (Fig. 29[7]); long pronotal carinae reach the mesoscutum; mesoscutum with few, scattered setae; notauli only insinuated; scutellar foveae completely defined with a transverse posterior carina inside (Fig. 31[7]); central part of metascutellum with imbricated sculpture. Male: unknown	***P.evenhuisi* (Pujade-Villar & Paretas-Martínez, 2006)**
6	Head, mesosoma and metasoma are yellowish brown	**7**
–	Head, mesosoma and metasoma are dark brown	**8**
7	Pedicel 1.5× as long as wide; F2 shorter than F3, F3–F10 subequal in length, width and shape (Fig. 29[29]); notauli deeply excavated; median mesoscutal impression not evident; rounded scutellar foveae but with a straight interior side; mesoscutum and scutellum have few scattered setae (Fig. 31[29]); mesoscutum and scutellum covered by scattered setae; propodeal carinae are independent and slightly curved in the last third; closed radial cell with all veins of the same thickness. Male: unknown	***P.xanthochroa* (Förster, 1869)**
–	F2–F10 subequal in length, width and shape (Fig. 29[21]); notauli deeply excavated with the median mesoscutal impression evident on the first two-thirds; large and oval scutellar foveae (Fig. 31[21]); mesoscutum and scutellum completely covered in long setae; central part of propodeum has few setae; propodeal carinae well-defined, straight and parallel; closed radial cell with the first half of R1 thinner than the second half only in females. Male: rhinaria and club shape begin at F2; F1 curved and longer than pedicel and F2, F2–F12 subequal; notauli is finer than in the females; veins of radial cell normal	***P.pilosus* (Andrews, 1978)**
8	Completely open radial cell; scutellar foveae absent	***P.indica* (Ferrer-Suay & Pujade-Villar, 2013)**
–	Closed radial cell; scutellar foveae present	**9**
9	Antennae longer than body	**10**
–	Antennae subequal or shorter than body	**16**
10	Rhinaria and club shape begin at F1	**11**
–	Rhinaria and club shape begin in other flagellomeres	**13**
11	Rhinaria and club shape begin in all parts of F1; F2 subequal to F3, F3 shorter than F4 (Fig. 29[17]); mesoscutum smooth and with few scattered setae that are only present on the anterior and lateral margins and few or none at the central part; scutellar foveae with a straight top and sides, open at the bottom (Fig. 31[17]); apex of scutellum with few setae; radial cell 2.7× as long as wide; Rs is slightly curved. Male: unknown	***P.longicornis* (Hartig, 1840)**
–	Rhinaria and club shape begin in last three-quarters of F1; different flagellomere proportions; mesoscutum has many scattered setae; different scutellar foveae; apex of scutellum has abundant setae; various radial cell sizes; Rs is straight	**12**
12	Last flagellomere 2.5× as long as wide (Fig. 29[27]); scutellar foveae has straight sides and open at top and bottom (Fig. 31[27]); propodeal carinae independent; radial cell 2.4× as long as wide. Male: unknown	***P.stricta* (Thomson, 1877)**
–	Last flagellomere 4.3× as long as wide (Fig. 29[13]); rounded scutellar foveae, slightly open at bottom (Fig. 31[13]); propodeal carinae joined at base; radial cell 2.9× as long as wide. Male: unknown	***P.insperatus* (Belizin, 1973)**
13	Rhinaria and club shape begin in same flagellomere	**14**
–	Rhinaria and club shape begin in different flagellomeres	**15**
14	Rhinaria and club shape begin at F2; pedicel shorter than F1, F1–F3 unequal in length (Fig. 29[26]); scutellar foveae slightly fused (Fig. 31[26]); propodeal carinae slightly curved; radial cell 2.9× as long as wide. Male: unknown	***P.stenos* (Andrews, 1978)**
–	Rhinaria and club shape begin at F3; pedicel longer than F1, F1–F3 subequal in length, (Fig. 29[11]); scutellar foveae not fused and open at top and bottom (Fig. 31[11]); propodeal carinae well-defined and straight; radial cell 2.7× as long as wide. Male: rhinaria and club shape begin at F3; F1 slightly curved and longer than pedicel and F2, F2 shorter than F3, F3 subequal to F4	***P.heterocera* (Hartig, 1841)**
15	Rhinaria begin at F1 and club shape begins at F2; F1 is 2.1× as long as pedicel (Fig. 29[2]); notauli clearly visible with two extensions at the base of mesoscutum just above foveae (Fig. 31[2]); longitudinal carinae present in metascutellum do not branch at base. Male: rhinaria and club shape begin at F2; F1 very curved and longer than pedicel and F2, F2 shorter than F3, F3 subequal to F4	***P.americana* (Baker, 1896)**
–	Rhinaria begin at F1 and club shape begins at F3; F1 is 1.3× as long as pedicel (Fig. 29[9]); notauli deeply excavated on the aterior part and weakly on the back, straight mesoscutum base (Fig. 31[9]); longitudinal carinae present in metascutellum branch at base. Male: rhinaria begins at F5 and club shape at F3; F2 subequal to F3, F3 shorter than F4	***P.fuscicornis* (Thomson, 1877)**
16	F1 longer than pedicel	**17**
–	F1 subequal or shorter than pedicel	**19**
17	Female: unknown. Male: rhinaria and club shape begin at F1; F2 thick and curved; F1 longer than pedicel and F2, F2–F4 subequal in length; scutellar foveae incomplete at top and bottom	***P.jeffersoni* (Ferrer-Suay & Pujade-Villar, 2014)**
–	Female: rhinaria and club shape begin at F3; different relations between flagellomeres; scutellar foveae completely defined or only slightly open on bottom. Male: unknown	**18**
18	F1 is 1.1× as long as pedicel, F1 longer than F2, F2 shorter than F3, F3–F4 subequal in length (Fig. 29[25]); mesoscutum with a line of setae next to each notaulus, notauli weakly present (Fig. 31[25]); scutellar foveae completely defined with two lines at top; propodeum has two short, straight carinae independently reach base. Male: unknown	***P.salicis* (Cameron, 1883)**
–	F1 is 1.3× as long as pedicel, F1 longer than F2, F2–F4 subequal in length (Fig. 29[10]); mesoscutum without setae in central part; notauli more marked on back than in front; scutellar foveae slightly open on bottom (Fig. 31[10]; propodeum with two well-defined carinae, slightly curved in last half. Male: unknown	***P.gutierrezi* (Andrews, 1978)**
19	Rhinaria begins at F2; F1 longer than F2, F2–F4 subequal in length; radial cell 2.7× as long as wide	***P.proximus* (Belizin, 1966)**
–	Rhinaria begins at F3; different flagellomere proportions; various radial cell sizes	**20**
20	Female: F1–F4 subequal in length (Fig. 29[18]); scutellar foveae rounded and separated by a thin carina (Fig. 31[18]); abundant setae on apex of scutellum; propodeum with narrow carinae (sometimes difficult to see). Male: rhinaria and club shape begin at F3; F1 not curved; F1 longer than pedicel and F2, F2 shorter than F3, F3–F4 subequal	***P.moldavica* (Ionescu, 1969)**
–	F1 longer than F2; without combination of characters as above	**21**
21	F2 shorter than F3, F3 subequal to F4 (Fig. 29[1]); scutellar foveae completely defined (Fig. 31[1]). Male: unknown	***P.abbreviata* (Thomson, 1877)**
–	F2 subequal to F3, F3 subequal or shorter than F4; scutellar foveae incomplete	**22**
22	F3 subequal to F4 (Fig. 29[15]); notauli clearly visible; scutellar foveae has superior and inferior margins that are not clearly delimited (Fig. 31[15])	***P.japonica* (Ferrer-Suay & Pujade-Villar, 2013)**
–	F3 shorter than F4; notauli present but slightly insinuated; scutellar foveae not delimited on bottom	***P.montoliui* (Ferrer-Suay & Pujade-Villar, 2013)**
23	Scutellar foveae not present	**24**
–	Scutellar foveae present, sometimes superficially	**29**
24	Open radial cell; body entirely covered by setae	**25**
–	Closed radial cell; body covered by scattered setae	**27**
25	Completely open radial cell that is 3.1× as long as wide	***P.chiangmaiensis* (Ferrer-Suay & Pujade-Villar, 2014)**
–	Partially open radial cell that can be different sizes	**26**
26	Rhinaria and club shape begin at F3 (Fig. 19[6]); radial cell 3.8× as long as wide (Fig. 31[6])	***P.china* (Ferrer-Suay & Pujade-Villar, 2013)**
–	Rhinaria and club shape begin at F2 (Fig. 19[3]); radial cell 2.6× as long as wide (Fig. 31[3])	***P.asiatica* (Ferrer-Suay & Pujade-Villar, 2013)**
27	Female: unknown. Male: rhinaria and club shape begin at F3; F1 long and curved; F2 slightly longer than F3, F3 subequal to F4; radial cell 3.1× as long as wide	***P.kenai* (Ferrer-Suay & Pujade-Villar, 2014)**
–	Female: rhinaria and club shape begin at F3 or F5; different sizes and ratios between flagellomeres; radial cell 2.5–2.6× as long as wide	**28**
28	Rhinaria and club shape begin at F5; F2 long and only slightly shorter than F1 (Fig. 29[16]); mesoscutum with abundant setae only in first half (Fig. 31[16]); mesopleural triangle open on anterior margin	***P.laevis* (Andrews, 1978)**
–	Rhinaria and club shape begin at F3 (sometimes difficult to see); F2 shorter than F1 (Fig. 29[19]); mesoscutum has setae present on anterior and lateral margins (Fig. 31[19]); complete mesopleural triangle	***P.nigripes* (Thomson, 1877)**
29	Radial cell partially open along anterior margin; F1 and F2 subequal; F1 curved in males (Fig. 29[28])	***P.villosa* (Hartig, 1841)**
–	Radial cell closed; F1 and F2 subequal or F1 longer than F2	**30**
30	Propodeal carinae form a plate	***P.wongchaiensis* (Ferrer-Suay & Pujade-Villar, 2014)**
–	Propodeal carinae are independent	**31**
31	Rhinaria and club shape begin at F2	**32**
–	Rhinaria and club shape begin at F3	**33**
32	Rounded scutellar foveae are separated by a carina and open at the bottom (Fig. 29[8]); scutellum has abundant setae; straight propodeal carinae are well-defined and join at the base; Rs slightly curved. Male: rhinaria and club shape begin at F2; F1 curved and longer than pedicel and F2, F2–F4 subequal	***P.falcata* (Andrews, 1978)**
–	Scutellar foveae practically absent (Fig. 29[20]); scutellum has few setae; propodeal carinae are slightly curved in the last third, clearly defined and reach the base independently. Male: unknown	***P.palmirae* (Pujade-Villar & Melika, 2018)**
33	Pedicel longer than F1, F1 longer than F2 (Fig. 30[1]); scutellar foveae almost completed (Fig. 32[1]); two propodeal carinae are well-defined at the top and undefined at the bottom; radial cell 2.7× as long as wide. Male: unknown	***P.belizini* (Pujade-Villar, 2018)**
–	Pedicel shorter than F1; different flagellomere proportions; mesoscutum has few scattered setae; incomplete scutellar foveae; two propodeal carinae that are well-defined; radial cell 2.7–2.8× as long as wide	**34**
34	F2 shorter than F3 (Fig. 29[5]); mesoscutum not gibbous; scutellum has few setae that are not abundant on the apex (Fig. 31[5]); propodeal carinae are slightly curved, well-defined at top and form a plate on bottom; radial cell 2.7× as long as wide. Male: F1 curved and longer than pedicel and F2, F2 subequal to F3	***P.calverti* (Andrews, 1978)**
–	F2 longer than F3 (Fig. 29[14]); mesoscutum characterised as very gibbous (Fig. 31[14]); scutellum with many setae, abundant on apex; propodeal carinae straight, well-defined and independently reaching base; radial cell 2.8× as long as wide. Male: unknown	***P.insularis* (Belizin, 1973)**

**Figure 29.**
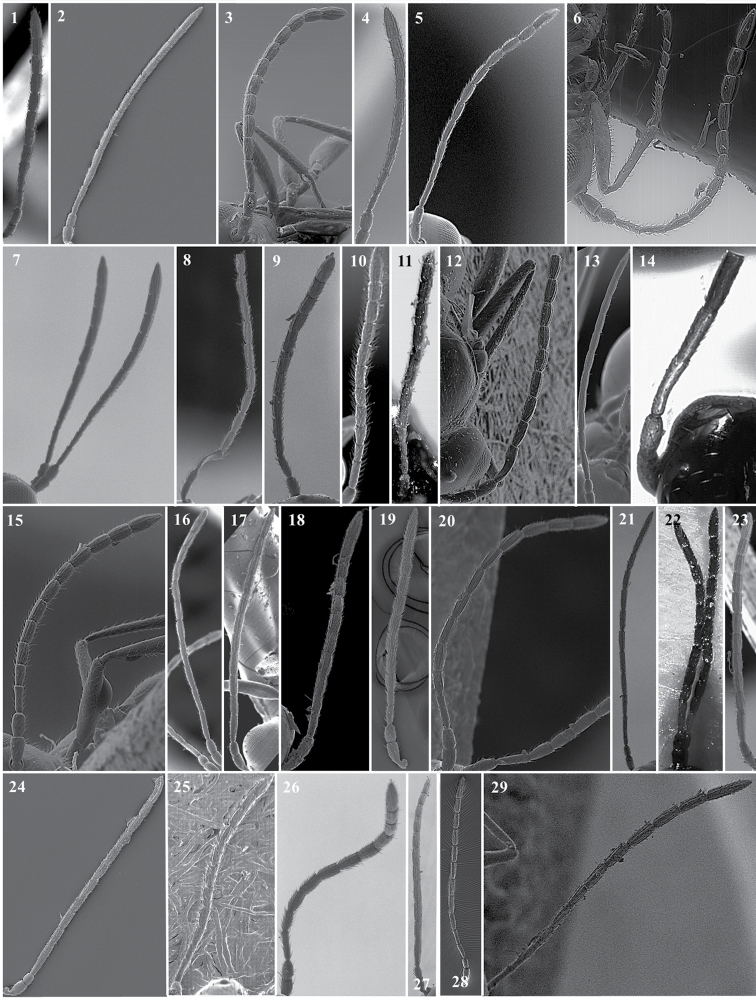
*Phaenoglyphis* antennae. *P.abbreviata* (**1**); *P.americana* (**2**); *P.asiatica* (**3**); *P.belizini* (**4**); *P.calverti* (**5**); *P.china* (**6**); *P.evenhuisi* (**7**); *P.falcata* (**8**); *P.fuscicornis* (**9**); *P.gutierrezi* (**10**); *P.heterocera* (**11**); *P.indica* (**12**); *P.insperatus* (**13**); *P.insularis* (**14**); *P.japonica* (**15**); *P.laevis* (**16**); *P.longicornis* (**17**); *P.moldavica* (**18**); *P.nigripes* (**19**); *P.palmirae* (**20**); *P.pilosus* (**21**); *P.proximus* (**22**); *P.pubicollis* (**23**); *P.ruficornis* (**24**); *P.salicis* (**25**); *P.stenos* (**26**); *P.stricta* (**27**); *P.villosa* (**28**); *P.xanthochroa* (**29**).

**Figure 30.**
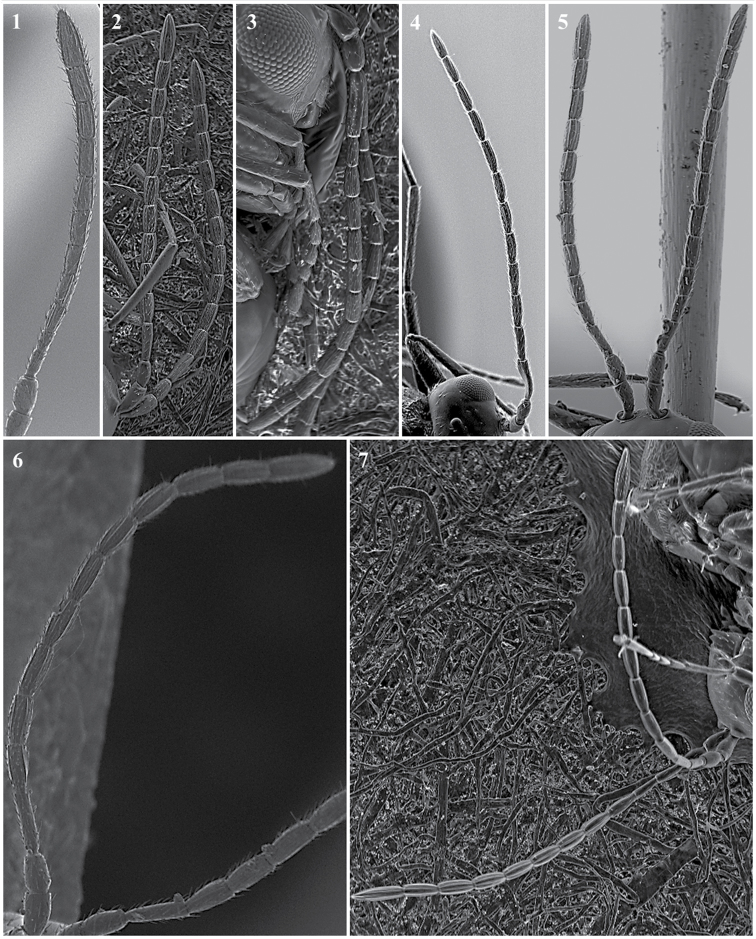
*Phaenoglyphis* antennae. *P.belizini* (**1**); *P.chiangmaii* (**2**); *P.jeffersonii* (**3**); *P.kenai* (**4**); *P.montoliu* (**5**); *P.palmirae* (**6**); *P.wongchaii* (**7**).

**Figure 31.**
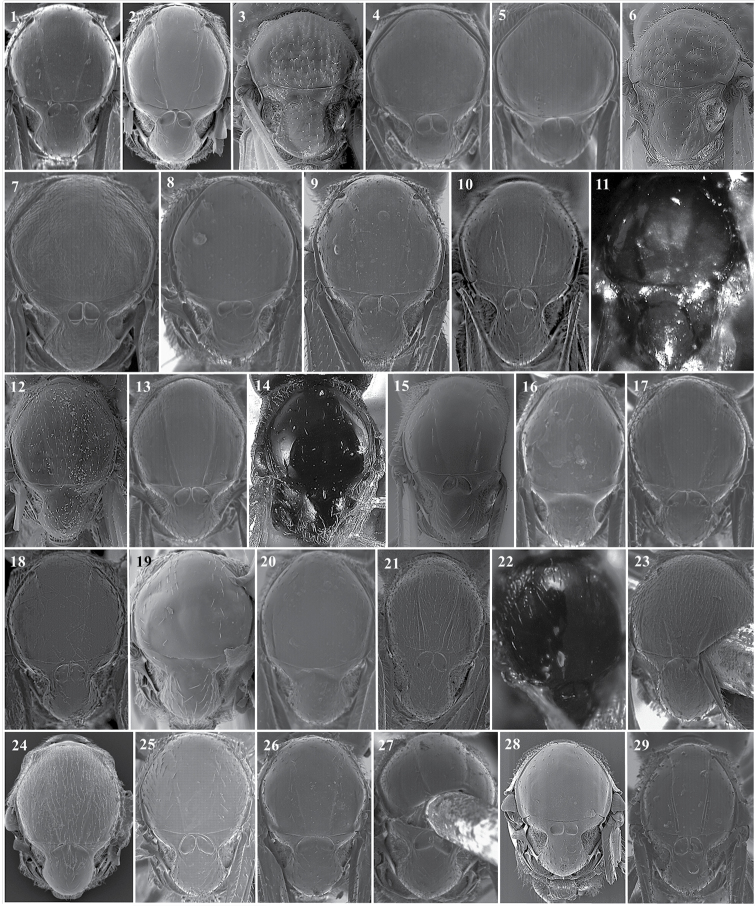
*Phaenoglyphis* mesoscutum. *P.abbreviata* (**1**); *P.americana* (**2**); *P.asiatica* (**3**); *P.belizini* (**4**); *P.calverti* (**5**); *P.china* (**6**); *P.evenhuisi* (**7**); *P.falcata* (**8**); *P.fuscicornis* (**9**); *P.gutierrezi* (**10**); *P.heterocera* (**11**); *P.indica* (**12**); *P.insperatus* (**13**); *P.insularis* (**14**); *P.japonica* (**15**); *P.laevis* (**16**); *P.longicornis* (**17**); *P.moldavica* (**18**); *P.nigripes* (**19**); *P.palmirae* (**20**); *P.pilosus* (**21**); *P.proximus* (**22**); *P.pubicollis* (**23**); *P.ruficornis* (**24**); *P.salicis* (**25**); *P.stenos* (**26**); *P.stricta* (**27**); *P.villosa* (**28**); *P.xanthochroa* (**29**).

**Figure 32.**
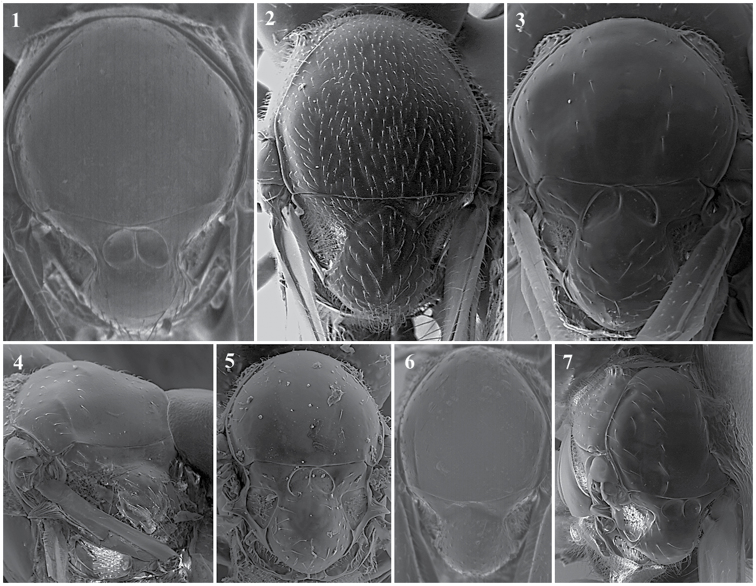
*Phaenoglyphis* mesoscutum. *P.belizini* (**1**); *P.chiangmaii* (**2**); *P.jeffersonii* (**3**); *P.kenai* (**4**); *P.montoliu* (**5**); *P.palmirae* (**6**); *P.wongchaii* (**7**).

#### Thoreauana

Taxon classificationAnimaliaHymenopteraFigitidae

Girault, 1930

Thoreauana Girault, 1930: 2. Type: Thoreauananativa Girault.

##### General features.

**Figure 33.**
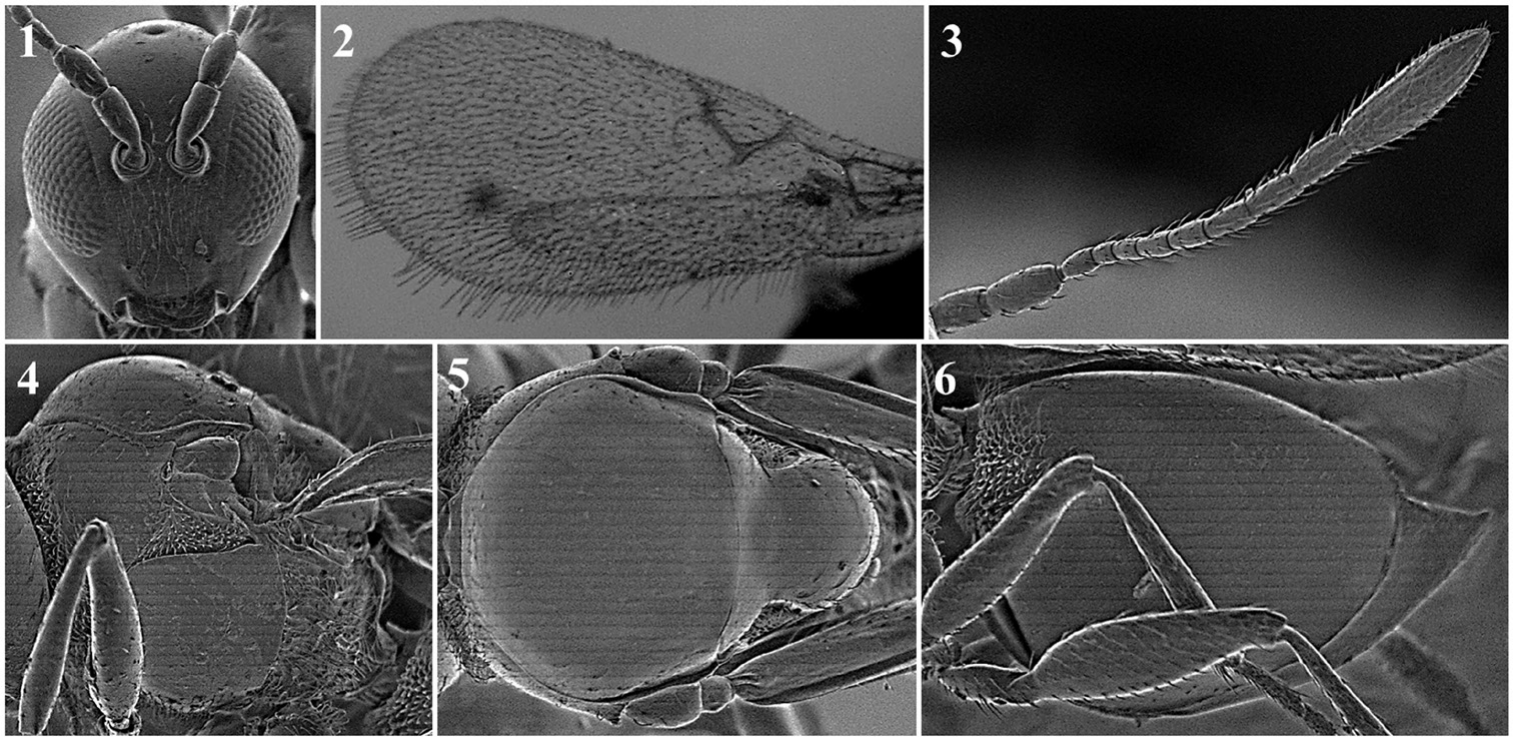
*Thoreauana* general features. Head (**1**); forewing (**2**); antenna (**3**); mesosoma (**4**); mesoscutum (**5**); metasoma (**6**).

*Head*. Rounded in anterior view, eyes at the midline of the head, malar space subequal to the distance from the external margin of the lateral ocellus to the dorsal margin of the compound eye, measured in the anterior view; abundant setae below the toruli; sparse setae on frons (Fig. 33[1]).

*Antenna*. Female: 11-segmented, clavate. Male: 12-segmented, clavate (Fig. 33[3]).

*Mesosoma*. Pronotum with setae only on its anterior part; pronotal carinae are small and only slightly indicated (Fig. 33[5]). Mesoscutum is smooth, shiny and almost without setae. No sutures on the mesopleuron (Fig. 33[4]). Scutellum smooth with sparse setae on its posterior and lateral parts; one small carina on each side of scutellum apex that are symmetrical, with a distance between them equivalent to the distance between the propodeal carinae. Propodeum with two strong and broad carinae (Fig. 33[5]).

*Forewing*. Large, longer than body, covered in dense pubescence; marginal setae present and long; veins yellow to light brown; radial cell small and completely open; R1 is very short, incomplete and does not reaching the costal margin; 2r is shorter than Sc + R1; Rs is short and nearly straight, reaching the wing margin; Cu1a, M + Cu1a, R s + M and M veins absent (Fig. 33[2]).

*Metasoma*. Proximal part has a complete ring of setae; metasoma non-segmented, only one tergite visible (Fig. 33[6]).

##### Distribution.

Australia (Girault 1930: 274, 1935: 2; Paretas-Martínez and Pujade-Villar 2006: 224).

##### Hosts.

Unknown.

### Key to species

**Table:** 

1	Female: unknown. Male: club shape begins at F3; F1 and F2 small and combined, shorter than pedicel and F3, F10 twice as long as other ﬂagellomeres, but not wider, forming a slender club (Fig. 34[1])	***T.giraulti* (Paretas-Martínez & Pujade-Villar, 2006)**
–	Club shape begins beyond F3; various size and combination of flagellomeres	**2**
2	Head with abundant, long setae on face (Fig. 34[4]); F1 shorter than pedicel but F1+ F2 longer than pedicel; F1–F3 long and subequal. Club shape begins at F4; F9 (F10 in males) forms a small club at the apex of the antenna; in females club dilated, in males club slender	***T.thoreauini* (Girault, 1935)**
–	Head with scattered setae on face. F1 shorter than pedicel and F1+F2 shorter than pedicel. F1–F3 very short and sometimes subequal. Club shape begin beyond F4	**3**
3	Female: F2 shorter than F1 and F3; club shape begins at F6. Male: F1–F3 subequal; club shape begins at F4 (Fig. 34[2])	***T.mascagnini* (Girault, 1935)**
–	Female: F1–F3 subequal; club shape begins at F8 (Fig. 34[3]). Male: unknown	***T.nativa* (Girault, 1930)**

**Figure 34.**
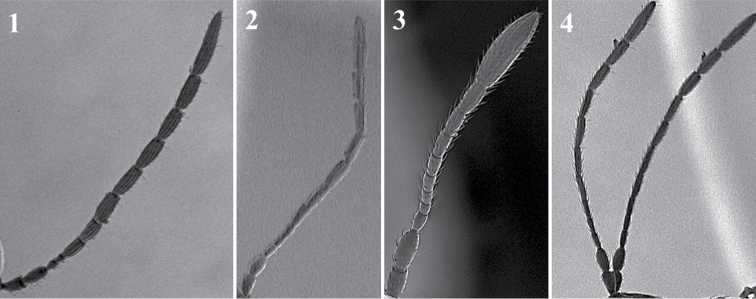
*Thoreauana* antennae. *T.giraulti* (**1**); *T.mascagnini* (**2**); *T.nativa* (**3**); *T.thoreauini* (**4**).

## Supplementary Material

XML Treatment for Alloxysta

XML Treatment for Apocharips

XML Treatment for Dilapothor

XML Treatment for Dilyta

XML Treatment for Lobopterocharips

XML Treatment for Lytoxysta

XML Treatment for Phaenoglyphis

XML Treatment for Thoreauana

## References

[B1] Andrews FG (1976) A new species of *Alloxysta* hyperparasitic on aphids associated with South American Nothofagus forest. Pan Pacific Entomologist 52(4): 256–257.

[B2] Andrews FG (1978) Taxonomy and host specificity of Nearctic Alloxystinae with a catalogue of the World species (Hymenoptera: Cynipidae). Occasional Papers in Entomology 25: 1–128.

[B3] Ashmead WH (1885) A bibliographical and synonymical catalogue of the North American Cynipidae, with descriptions of new species. Transactions of the American Entomological Society 12: 291–304.

[B4] Ashmead WH (1896) Descriptions of new parasitic Hymenoptera. Transactions of the American Entomological Society 12: 291–304.

[B5] Ashmead WH (1900) Notes on some New Zealand and Australian parasitic Hymenoptera, with descriptions of new genera and new species. Proceedings of the Linnean Society of New South Wales 25: 327–360. 10.5962/bhl.part.12157

[B6] Ashmead WH (1903) Classification of the gall-wasps and the parasitic Cynipoids, or the superfamily Cynipoidea III. Psyche 10: 140–155. 10.1155/1903/83423

[B7] Ashmead WH (1904) Descriptions of new Hymenoptera from Japan. J.N.Y. Entomological Society 12: 65–84.

[B8] Baker CF (1896) New American parasitic Cynipidae (Allotriinae). Canadian Entomologist 28: 131–135. 10.4039/Ent28131-5

[B9] Belizin VI (1962) New Parasitoid Cynipoidea species (Hymenoptera) from a Far East. Communications of the Far East Branch of the Russian Academy os Sciences (Siberian Section) 16: 125–129.

[B10] Belizin VI (1966) Paraziticheskie tsinipidy (Hymenoptera, Cynipoidea) moldavskoj SSR (Parasitic Cynipids (Hymenoptera, Cynipoidea) in the Mmoldavian SSR). Trudy Moldavskoho nauchono-issled. Instituta Sadovodstva, Vinogradarstva i Vinodelija (Entomologia) 13: 1–14.

[B11] Belizin VI (1968) New genera and species of gall wasps (Hymenoptera, Cynipoidea) of the Soviet far east and adjacent territories. District Station of Plant Protection (Kursk) 5: 701–719.

[B12] Belizin VI (1973) New Cynipids (Hymenoptera, Cynipoidea) from the USSR and Neighbouring countries. Revue d’Entomologie de l’URSS 52(1): 29–38.

[B13] Benoit PLG (1956) Deux Cynipidae-Charipinae inedits du Congo Belge. Revue de zoologie et de botanique africaines 53: 437–440.

[B14] Brèthes J (1913) Description d’un nouveau genre et d’une nouvelle espece de Cynipide du Chili. Boletin del Museo Nacional 5(1): 200–201.

[B15] Cameron P (1879) On some new or little known British Hymenoptera. Transactions of the Entomological Society of London 1879: 107–119.

[B16] Cameron P (1883) Descriptions of sixteen new species of parasitic Cynipidae, chiefly from Scotland. Transactions of the Entomological Society of London 16(4): 365–374. 10.1111/j.1365-2311.1883.tb02952.x

[B17] Cameron P (1886) The fauna of Scotland, with special referenctasche to Clydesdale and the western district. Proceedings of the Natural History Society of Glasgow 3: 53–95.

[B18] Cameron P (1889) On the British species of Allotrinae, with descriptions of other new species of parasitic Cynipidae. Memoirs of Manchester Literary and Philosophical Society 2: 53–69.

[B19] Carver M (1992) Alloxystinae (Hymenoptera, Cynipoidea, Charipidae) in Australia. Invertebrate Taxonomy 6(3): 769–785. 10.1071/IT9920769

[B20] Curtis J (1838) British entomology; being illustrations and descriptions of the genera of insects found in Great Britain and Ireland: containing coloured figures of naturae of the rarest, and beautiful species and in many instances of the plants upon which their are found. Privately published (London) 15: 674–721.

[B21] Evenhuis HH (1973) Studies on CynipidaeAlloxystinae. 3. The identity of *Phaenoglyphisruficornis* (Förster, 1869) comb. nov. Entomologische Berichten 33: 218–219.

[B22] Fergusson NDM (1986) Charipidae, Ibaliidae and Figitidae (Hymenoptera: Cynipoidea). Handbook of Identification British Insects 8(1c): 1–55.

[B23] Ferrer-Suay M Selfa J Pujade-Villar J (2011) Nuevos registros de la subfamilia Charipinae (Hymenoptera, Cynipoidea, Figitidae) para Andorra junto con una clave identificativa. Boletín de la Asociación Española de Entomología 35(3–4): 345–367.

[B24] Ferrer-Suay M Paretas-Martínez J Selfa J Pujade-Villar J (2012a) Taxonomic and synonymic world catalogue of the Charipinae and notes about this subfamily (Hymenoptera: Cynipoidea: Figitidae). Zootaxa 3376: 1–92.

[B25] Ferrer-Suay M Selfa J Pujade-Villar J (2012b) Charipinos de Colombia (Hymenoptera: Figitidae), con la descripción de dos nuevas especies. Revista Colombiana de Entomología 38(2): 320–328.

[B26] Ferrer-Suay M Paretas-Martínez J Selfa J Pujade-Villar J (2012c) Charipinae fauna from New Zealand with descriptions of two new species of *Alloxysta* Förster (Hymenoptera: Cynipoidea: Figitidae: Charipinae). Australian Journal of Entomology 51: 229–238. 10.1111/j.1440-6055.2012.00859.x

[B27] Ferrer-Suay M Selfa J Pujade-Villar J (2012d) First record of *Alloxysta* Förster from Madagascar, with description of two new species (Hymenoptera: Cynipoidea: Figitidae: Charipinae). African Entomology 20(2): 222–228. 10.4001/003.020.0208

[B28] Ferrer-Suay M Selfa J Tomanović Z Janković M Kos K Rakhshani E Pujade-Villar J (2013a) Revision of *Alloxysta* from the north-western Balkan Peninsula with description of two new species (Hymenoptera: Figitidae: Charipinae). Acta Entomologica Musei Nationalis Pragae 53(1): 347–368.

[B29] Ferrer-Suay M Selfa J Equihua-Martínez A Estrada-Venegas E Lomeli-Flores R Peña Martínez R Pujade-Villar J (2013b) Charipinae (Hymenoptera: Cynipoidea: Figitidae) from Mexico with description of three new species. Annals of the Entomological Society of America 106(1): 26–41. 10.1603/AN12022

[B30] Ferrer-Suay M Paretas-Martínez J Pujade-Villar J (2013c) Revision of *Apocharips* Fergusson (Hymenopterza: Figitidae: Charipinae) with description of three new species from Colombia. Zootaxa 3646(4): 487–500. 10.11646/zootaxa.3646.4.8 26213775

[B31] Ferrer-Suay M Selfa J Pujade-Villar J (2013d) Revision of Thomson and Zetterstedt collections of *Alloxysta* genus deposited in Lund Museum of Zoology (Sweden). Entomologisk Tidskrift 134: 77–102.

[B32] Ferrer-Suay M Selfa J Notton D Pujade-Villar J (2013e) Revision of the types of species of *Alloxysta* Förster, 1869 described by Cameron and Fergusson (Hymenoptera: Figitidae: Charipinae) deposited in the Natural History Museum (London) including a key to the fauna of Great Britain. European Jounal of Taxonomy 53: 1–27. 10.5852/ejt.2013.53

[B33] Ferrer-Suay M Selfa J Pujade-Villar J (2013f) Revision of *Alloxysta* from the Curtis collection (Hymenoptera: Figitidae: Charipinae) deposited in Museum Victoria (Australia). Memoirs of Museum Victoria 70: 11–16. 10.24199/j.mmv.2013.70.02

[B34] Ferrer-Suay M Selfa J Pujade-Villar J (2013g) The *Alloxysta* (Hymenoptera: Figitidae: Charipinae) type material in the United States National Museum of Natural History and the Canadian National Collection of Insects. The Canadian Entomologist 145(6): 603–625. 10.4039/tce.2013.52

[B35] Ferrer-Suay M Paretas-Martínez J Pujade-Villar J (2013h) Revision of *Apocharips* Fergusson (Hymenopterza: Figitidae: Charipinae) with description of three new species from Colombia. Zootaxa 3646(4): 487–500. 10.11646/zootaxa.3646.4.8 26213775

[B36] Ferrer-Suay M Selfa J Seco MV Pujade-Villar J (2014a) Revision of Hellén types of *Alloxysta* Förster (Hymenoptera: Figitidae, Charipinae). Entomologica Fennica 25: 86–101.

[B37] Ferrer-Suay M Selfa J Pujade-Villar J (2014b) Review of the Hartig type collection of *Alloxysta* (Hymenoptera: Figitidae: Charipinae) and other *Alloxysta* material deposited in the Zoologische Staatssammlung Museum (Munich). Fragmenta Faunística 57(2): 75–116. 10.3161/00159301FF2014.57.2.075

[B38] Ferrer-Suay M Selfa J Pujade-Villar J (2015) New contribution to the knowledge of the genus *Alloxysta* (Hymenoptera: Cynipoidea: Figitidae): revision of some type material. Annalen des Naturhistorischen Museums in Wien, Serie B 117: 23–36.

[B39] Ferrer-Suay M Selfa J Mata-Casanova N Perez Hidalgo N Pujade-Villar J (2018) Worldwide revision of the genus *Phaenoglyphis* Förster, 1869 (Hymenoptera, Cynipoidea, Figitidae, Charipinae). Insect Systematics & Evolution 1–62. 10.1163/1876312X-00002177

[B40] Förster A (1869) Ueber die Gallwespen. Verhandlungen der Zoologisch-Botanischen Gesellschaft in Wien 19: 327–370.

[B41] Girault AA (1930) New pests from Australia, VIII. Privately published. Brisbane, Australia, 6 pp.

[B42] Hartig T (1840) Ueber die Familie der Gallwespen. Zeitschrift für Entomologie (Germar) 2: 176–210.

[B43] Hartig T (1841) Erster nachtrag zur naturgeschichte der Gallwespen. Zeitschrift für Entomologie (Germar) 3: 322–358.

[B44] Hellén W (1963) Die Alloxystininen Finnlands (Hymenoptera: Cynipidae). Fauna Fennica 15: 1–23.

[B45] Hübner J (1816–1825) Verzeichniss bekannter Schmetterlinge, Augsburg, 431 + 72 pp.

[B46] Ionescu MA (1969) HymenopteraCynipoidea. In: Fauna Republicii Socialiste România, 9, 6. Editura Academiei Republicii Socialiste România, Bucureşti, 285 pp.

[B47] Kieffer JJ (1900) Ueber Allotrinen. Wiener Entomologische Zeitung 19: 112–115. 10.5962/bhl.part.3441

[B48] Kieffer JJ (1902a) Description de quelques Cynipides nouveaus ou peu connus et de deux de leurs parasites (Hymenopteres). Bulletin de la Société d’Histoire Naturelle de Metz 10: 1–18.

[B49] Kieffer JJ (1909) Beschreibung neuer in Blattlausen schmartozender Cynipiden. Naturwissenschaftliche Zeitschrift für Forsten und Landwirtschaft Stuttgart 7: 479–482.

[B50] Kierych E (1988) A new genus and a new species of cynipoids (Hymenoptera, Cynipoidea, Charipidae) from Poland. Annales Zoologici 41: 351–354.

[B51] Kovalev OV (1994) Paleontological history, phylogeny and the system of Brachy-cleistogastromorphs and Cynipomorphs (Hymenoptera, Brachycleistogastro-morpha infraorden N., Cynipomorpha infraorden N.) with description of new fossil and recent families, subfamilies and genera). Entomologicheskoye Obozreniye 73(2): 385–426.

[B52] Marshall TA (1870) On some British Cynipidae. Entomol 6: 178–181.

[B53] Menke AS (1993) A new species of *Apocharips* from Costa Rica (Hymenoptera: Cynipoidea, Charipidae). Journal of Hymenoptera Research 2(1): 97–100.

[B54] Menke AS Evenhuis HH (1991) North American Charipidae: key to genera, nomenclature, species checklists, and a new species of *Dilyta* Förster (Hymenoptera: Cynipoidea). Proceedings of the Entomological Society of Washington 93: 136–158.

[B55] Paretas-Martínez J Pujade-Villar J (2005) First Record of Charipinae from Taiwan: *Alloxystamara* sp. nov. (Hymenoptera: Cynipoidea: Figitidae). Zoological Studies 44(4): 458–461.

[B56] Paretas-Martínez J Pujade-Villar J (2006) Two genera of Charipinae (Hymenoptera: Figitidae) from Australia: revision of the genus *Thoreauana* Girault, 1930 and description of *Dilapothor* n. gen. Australian Journal of Entomology 45: 219–226. 10.1111/j.1440-6055.2006.00536.x

[B57] Paretas-Martínez J Pujade-Villar J (2007) Revisión de los Charipinae de la región Neotropical (Hymenoptera: Figitidae). Entomología mexicana 6(2): 1344–1348.

[B58] Paretas-Martínez J Arnedo MA Melika G Selfa J Seco-Fernández MV Fülöp D Pujade-Villar J (2007) Phylogeny of the parasitic wasp subfamily Charipinae (Hymenoptera, Cynipoidea, Figitidae). Zoologica Scripta 36: 153–172. 10.1111/j.1463-6409.2006.00269.x

[B59] Paretas-Martínez J Melika G Pujade-Villar J (2009) Description of four new species of *Dilyta* Förster (Hymenoptera: Figitidae: Charipinae) from the Afrotropical Region. African Entomology 17(2): 207–214. 10.4001/003.017.0211

[B60] Paretas-Martínez J Ferrer-Suay M Kovalev O Melika G Selfa J Pujade-Villar J (2011) Revision of the species of *Dilyta* Förster (Hymenoptera: Figitidae: Charipinae) present in the holarctic, with description of four new species from the eastern palaearctic. Zootaxa 2780: 29–38.

[B61] Pujade-Villar J Ferrer-Suay M (2011) First records of genus Dilyta from Madagascar with description of *Dilytaparetasmartinezi* n. sp. (Hymenoptera: Cynipoidea: Figitidae: Charipinae). Orsis 26: 139–144.

[B62] Pujade-Villar J Díaz N Evenhuis HH Ros-Farré P (2002) South American Charipinae: Review and description of two new species (Hymenoptera: Cynipoidea: Figitidae). Annals of the Entomological Society of America 95(5): 541–546. 10.1603/0013-8746(2002)095[0541:SACRAD]2.0.CO;2

[B63] Quinlan J Evenhuis HH (1980) Status of the subfamily names Charipinae and Alloxystinae (Hymenoptera: Cynipidae). Systematical Entomology 5: 427–430. 10.1111/j.1365-3113.1980.tb00426.x

[B64] Rohwer SA Fagan M (1919) Additions and corrections to “The type-species of the genera of the Cynipoidea, or the gall wasps and parasitic Cynipoids”. Proceedings of the United States National Museum 55: 337–340. 10.5479/si.00963801.2266.237

[B65] Schoenherr CJ (1826) Curculionodum Dispositio methodica cum generum characteribus, descriptionibus atque observationibus variis, seu prodromus ad Synonymiae Insectorum 4. Lipsiae, 338 pp.

[B66] Thomson CG (1862) Forsok till uppstallning och beskrifning af Sveriges Figiter. Öfversigt af Kongl. Svenska Vetenskaps-Akad: s förhandl 18: 395–420.

[B67] Thomson CG (1877) Ofversikt af Sveriges Cynips-arter. Opuscula Entomologica 8: 778–820.

[B68] Westwood JO (1833) Notice of the habits of a Cynipidous insect parasitic upon the *Aphisrosae* with descriptions of several other parasitic Hymenoptera. Magazine of Natural History 6: 491–497.

[B69] Zetterstedt JW (1838) Insecta Lapponica descripta: Hymenoptera. Voss, Lipsiae, 315–476.

